# Convergent Comodulation Reduces Interindividual Variability of Circuit Output

**DOI:** 10.1523/ENEURO.0167-24.2024

**Published:** 2024-09-03

**Authors:** Anna C. Schneider, Elizabeth Cronin, Nelly Daur, Dirk Bucher, Farzan Nadim

**Affiliations:** NJIT, Newark, New Jersey 07102

**Keywords:** central pattern generator, neuromodulation, stomatogastric, variability

## Abstract

Ionic current levels of identified neurons vary substantially across individual animals. Yet, under similar conditions, neural circuit output can be remarkably similar, as evidenced in many motor systems. All neural circuits are influenced by multiple neuromodulators, which provide flexibility to their output. These neuromodulators often overlap in their actions by modulating the same channel type or synapse, yet have neuron-specific actions resulting from distinct receptor expression. Because of this different receptor expression pattern, in the presence of multiple convergent neuromodulators, a common downstream target would be activated more uniformly in circuit neurons across individuals. We therefore propose that a baseline tonic (non-saturating) level of comodulation by convergent neuromodulators can reduce interindividual variability of circuit output. We tested this hypothesis in the pyloric circuit of the crab, *Cancer borealis*. Multiple excitatory neuropeptides converge to activate the same voltage-gated current in this circuit, but different subsets of pyloric neurons have receptors for each peptide. We quantified the interindividual variability of the unmodulated pyloric circuit output by measuring the activity phases, cycle frequency, and intraburst spike number and frequency. We then examined the variability in the presence of different combinations and concentrations of three neuropeptides. We found that at mid-level concentration (30 nM) but not at near-threshold (1 nM) or saturating (1 µM) concentrations, comodulation by multiple neuropeptides reduced the circuit output variability. Notably, the interindividual variability of response properties of an isolated neuron was not reduced by comodulation, suggesting that the reduction of output variability may emerge as a network effect.

## Significance Statement

Neuromodulation has been explored as a mechanism to provide flexibility to the output of neural circuits. All neural circuits are subject to neuromodulation by multiple substances. These multiple neuromodulators often have convergent subcellular actions, and yet different circuit neurons express receptors for different neuromodulators. This pattern of cellular-level convergence and circuit-level divergence gives rise to the possibility that the presence of many modulators at subsaturating concentrations may provide a consistent level of modulatory action of the circuit without qualitatively altering this output. This possibility indicates a different but complementary role for neuromodulation: that convergent comodulation at sub-saturation levels reduces interindividual variability of neural circuit output.

## Introduction

Historically, interindividual differences in animal behavior and in the function of neural circuits that underlie these behaviors have been disregarded, and most studies have examined the average behavioral response and the mean activity of the neural circuits involved (Asahina et al., 2022). Yet, recent studies have shown that behaviors as simple as reflexes or as complex as physiological responses to psychedelics can vary significantly among individuals (Cerins et al., 2022; Moujaes et al., 2023; Xu et al., 2022), even when accounting for genotype and other variables (Hageter et al., 2021; Palavicino-Maggio and Sengupta, 2022; Rihani and Sachse, 2022). Behavioral variability results largely from variability of underlying neural circuits (Feierstein et al., 2015; Rihani and Sachse, 2022; Tamvacakis et al., 2022), and such variability is present even in smaller circuits in invertebrate animals (Norris et al., 2011; Wenning et al., 2014, 2018; Goaillard and Marder, 2021). Despite significant variability, a neural circuit can generate an output good enough to produce the related behavior (Nassim, 2018); yet, in most cases, the CNS precisely controls behavior and significant variability results in dysfunctional output. Thus, at some level, circuit output must be constrained enough to produce consistent and meaningful behavior.

Several factors have been proposed to promote a consistent output at the level of an individual neuron. These include correlations of ion channel expression (Khorkova and Golowasch, 2007; Tobin et al., 2009; Temporal et al., 2011; Golowasch et al., 2017; Tran et al., 2019), output degeneracy (Ransdell et al., 2013; Goaillard and Marder, 2021), and excitatory neuromodulation (Schneider et al., 2022). Whether any of these mechanisms results in reduction of interindividual variability at the circuit level remains unexplored.

Neuromodulation is conventionally thought to provide flexibility in neural circuit operation (Nadim and Bucher, 2014). Yet, at a single-neuron level, excitatory neuromodulation has been shown to substantially reduce interindividual variability due to increasing excitability threshold and relatively consistent saturation levels (Schneider et al., 2022). At the circuit level, however, the effect of any neuromodulator is directly on the subset of neurons that express receptors for that modulator and, indirectly, through the dynamics that arise from the interactions of neurons. Therefore, it is not easy to intuit how the reduction of variability of neuronal function translates to variability of circuit activity. In addition, neuromodulators do not act independently, and, at any time, neural circuits are comodulated by multiple substances (Harris-Warrick, 2011; Marder, 2012; Russo, 2017). Different comodulators acting on a circuit can have distinct, overlapping, or convergent actions. For instance, in the crustacean stomatogastric system, monoamines have distinct effects on various aspects of excitability and synaptic output in different neuron types (Harris-Warrick, 2011). In contrast, multiple excitatory neuropeptides and muscarinic agonists have consistent actions across neuron types (Marder and Bucher, 2007). Consequently, different modulators often share subcellular targets, while remaining distinct in receptor expression levels or circuit neuron targets. Based on these facts, we propose an additional role for neuromodulation: that baseline (tonic) comodulation by multiple neuromodulators that have convergent effects, but different neural targets or patterns of receptor expression, could produce an averaged consistent effect on all targeted neurons and could thereby promote a consistent circuit output across individuals.

We tested this hypothesis in the pyloric network of the stomatogastric ganglion (STG) of the crab *Cancer borealis*, which has been a long-standing testbed for neuromodulation (Stein, 2009; Marder et al., 2014; Daur et al., 2016; Dickinson and Powell, 2023). The pyloric circuit consists of identified neurons with known synaptic connections. Under physiological conditions, it expresses regular periodic activity (∼1 Hz) that can be easily quantified. Upon removal of all neuromodulatory inputs (decentralization), the pyloric rhythm is disrupted or significantly slowed and shows significant interindividual variability (Hamood et al., 2015). We measured several circuit activity attributes (cycle frequency, activity phases, etc.) after decentralization and in the presence of one, two, or three neuropeptides, applied at the same total concentration. We then quantified the effect of comodulation on the interindividual variability of these attributes. To see if the change in variability with increasing numbers of peptides is consistent at the circuit level and the single-neuron level, we used the same peptide combinations to assess the activity attributes of a single synaptically isolated pyloric neuron.

## Materials and Methods

### Experimental preparation

**Figure 1.**
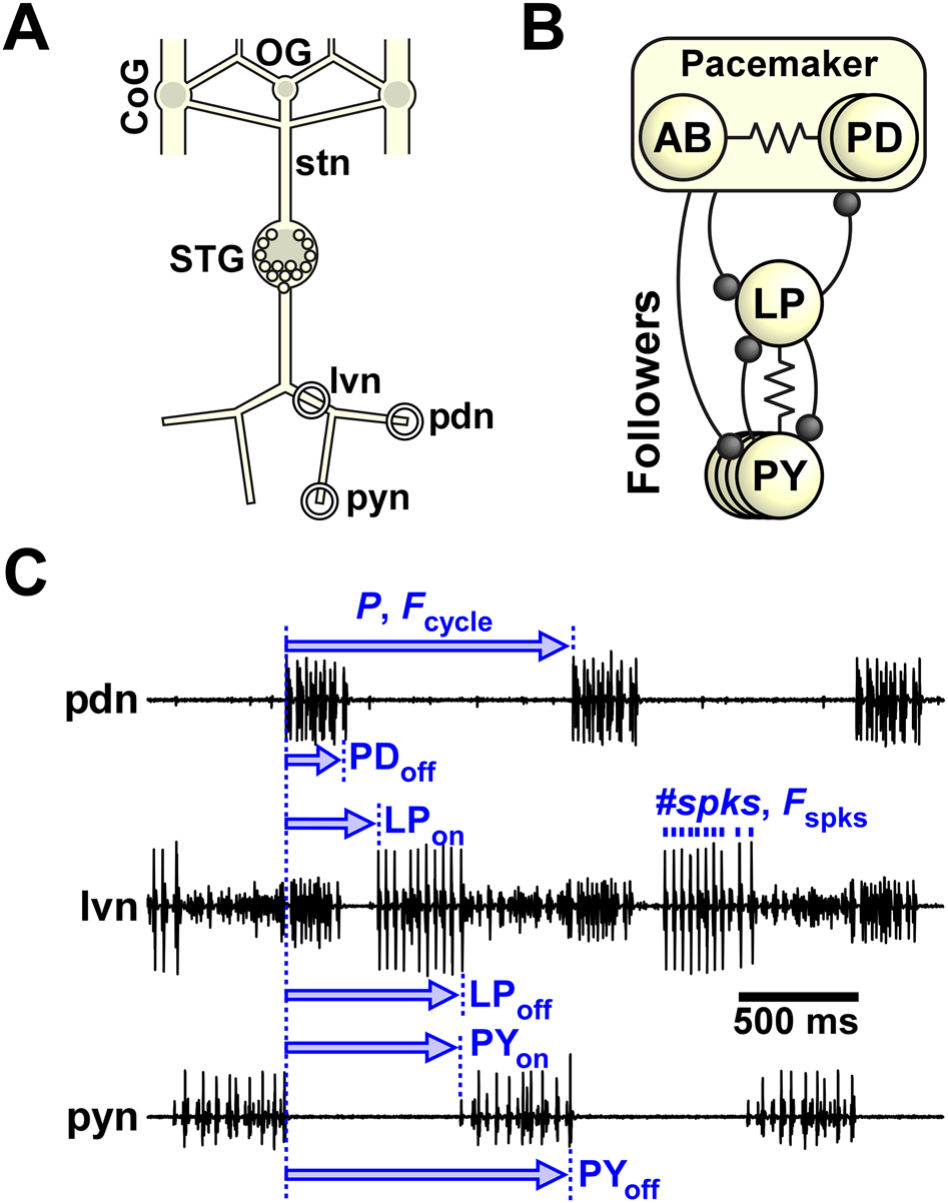
The stomatogastric nervous system (STNS) of crabs. ***A***, Schematic of the STNS of the crab *C. borealis*. Neurons of the pyloric circuit are located in the stomatogastric ganglion (STG). They receive input from neuromodulatory projection neurons that originate in the paired commissural ganglia (CoG) and the unpaired esophageal ganglion (OG) and send their axons through the stomatogastric nerve (stn). Pyloric motor neurons project their axons through the lateral ventricular (lvn), pyloric dilator (pdn), and pyloric constrictor (pyn) nerves. The extracellular recording sites are indicated by circles. ***B***, The core pyloric circuit consists of a group of pacemaker neurons: the anterior burster (AB) and two pyloric dilator (PD) neurons. They inhibit their followers, the lateral pyloric (LP) and 3–5 pyloric constrictor (PY) neurons. Inhibitory synapses are shown as circles, and electrical connections are depicted with resistor symbols. ***C***, The pyloric rhythm and rhythm parameters. The activity of the PD neurons from the pacemaker group is recorded from the pdn. Activity of all neurons can be recorded from the lvn, where LP is typically the largest unit, PD is mid-sized, and PY are the smallest units. In addition, PD and PY activity are also recorded from the pdn and pyn, respectively. Cycle period is defined as the time from the beginning of one PD burst (PD_on_) to the next. *F*_cycle_ is the inverse of cycle period (*P*). Burst start (on) and end (off) for each neuron is calculated by dividing the latency with respect to PD_on_ by the cycle period. In addition, we counted the number of spikes per burst (#spks) and calculated the average spike frequency (*F*_spks_) by dividing #spks-1 by burst duration (off-on).

Experiments were performed on the isolated stomatogastric nervous system (STNS) of male Jonah crabs (*C. borealis*) obtained from local fish markets and maintained in recirculating artificial seawater tanks at 12°C with a 12 h light/dark cycle until use. Crabs were anesthetized by chilling in ice for at least 30 min before removing the stomach. The STNS was carefully dissected from the stomach and pinned out dorsal side up in a Sylgard (Dow Corning) lined petri dish. All STNS preparations consisted of the stomatogastric ganglion (STG), the anterior portion that contains modulatory projection neurons, and motor nerves including the lateral ventricular (lvn), pyloric dilator (pdn), and pyloric constrictor (pyn) nerves (Fig. 1*A*). The sheath around the STG was removed with fine tungsten pins to facilitate intracellular recording and the penetration of bath-applied chemicals. A large petroleum jelly well was constructed around the STG to allow for fast exchange of solutions and was constantly perfused with cold saline (11–12°C). Smaller wells for extracellular recordings were constructed around the lvn, pdn, and pyn. To remove the influence of neuromodulatory projection neurons on the STG neurons, the stomatogastric nerve (stn) was transected with fine scissors (decentralization).

### Solutions

*C. borealis* saline contained (in mM): 440 NaCl, 26 MgCl_2_, 13 CaCl_2_, 11 KCl, 10 Tris base, 5 maleic acid, buffered to pH 7.4. Proctolin (PROC; RS Synthesis), crustacean cardioactive peptide (CCAP; RS Synthesis), and red pigment concentrating hormone (RPCH; GenScript) were prepared as 10^−3^ M aliquots (PROC and CCAP in distilled water, RPCH in dimethyl sulfoxide: DMSO, Fisher Scientific) and stored at −20°C until use. The stock solutions were thawed and diluted to a final concentration in either normal saline or saline with 10^−5^ M picrotoxin (PTX, Sigma-Aldrich), immediately before the experiments. The final total modulator concentrations were 10^−9^ M (low), 3 × 10^−8^ M (mid), and 10^−6^ M (high). In a few initial experiments, a concentration of 10^−8^ M was used as mid. The total modulator concentration (low, mid, or high) was held constant in comodulator applications so that with two modulators each was one-half of the total concentration and with three modulators each was one-third of the total concentration. For simplicity of notation, we will use the following single-letter abbreviations in the manuscript: P = PROC, C = CCAP, and R = RPCH. When we use more than one modulator, we will use combinations of these single letters, such as PC = PROC + CCAP. Therefore, for example, the notation “low PCR” indicates the application of PROC + CCAP + RPCH, each at (1/3) × 10^−9^ M.

For experiments that assessed single-neuron excitability, synapses were blocked with PTX. PTX was dissolved in DMSO at 10^−2^ M and stored as stock solution at 4°C. Immediately before the experiment, PTX stock solution was diluted to a final concentration of 10^−5^ M in saline.

### Electrophysiology

Thin stainless steel pin electrodes were placed inside and outside petroleum jelly wells around the lvn, pdn, and pyn to record the pyloric rhythm extracellularly. The extracellular electrodes were connected to a differential AC amplifier (Model 1700, A-M Systems). To reach a steady state in each neuromodulatory condition, we waited 20–30 min after decentralization before washing in any neuromodulator, and 10–15 min before switching from one neuromodulator cocktail to the next. For analysis, we recorded the pyloric rhythm for 1–2 min in steady state in the intact STNS, after decentralization (ctrl), and after washing in each of the sequence of neuropeptides applied at different concentrations and combinations. In total, our experiments were done on the following datasets:

1. Intact → ctrl → low P, PC, PCR → mid P, PC, PCR → wash,
2. Intact → ctrl → mid P, PC, PCR → wash,
3. Intact → ctrl → high P or high PC.

Because modulators were added sequentially, we did not wash out with saline between modulatory conditions. To minimize the variability that can be caused by changing environmental or experimental factors, we typically did these experiments simultaneously with two preparations in the same dish and at the same time of the day.

To examine the variability of intrinsic response properties, we used intracellular two-electrode current-clamp recordings. Intracellular voltage recording and current injection were done with Axoclamp 900A amplifiers (Molecular Devices). All recordings were digitized at 5 kHz (Digidata 1440A, Molecular Devices) and recorded with Clampex 10.6 (Molecular Devices).

We first identified the lateral pyloric (LP) and the two pyloric dilator (PD) neurons in an intact preparation by matching the intracellular activity to the extracellular activity on the lvn and pdn and by their characteristic intracellular membrane potential waveforms. We then decentralized the preparation, impaled the LP neuron with two electrodes and both PD neurons with one electrode each, and washed in PTX for at least 15 min until inhibitory postsynaptic potentials in the PD neuron (from the presynaptic LP neuron) disappeared and LP inhibition during PD bursts was greatly reduced. We then ran current-clamp protocols as described in Schneider et al. (2022) in the LP neuron while hyperpolarizing the PD neurons with −5 nA DC current to eliminate the remaining PTX-insensitive PD to LP synaptic inhibition. Each set of protocols was repeated in decentralized, mid P, mid PC, mid PCR, and wash. To obtain *f–I* curves, we depolarized LP by injecting current steps from 0 to 5 nA, in increments of 0.5 nA. To measure *f–I* hysteresis, we also used the inverse sequence, from 5 to 0 nA. We ran each protocol twice and used the average spike responses for analysis (see below). To measure rebound properties, we used two protocols. First, we hyperpolarized LP five times with 10 s current steps of −5 nA, interspersed with 10 s recovery time. This interval was long enough for LP to return to initial conditions before the next sweep (Schneider et al., 2022). Second, we hyperpolarized LP periodically with 20 1 s on/1 s off, −5 nA current steps to mimic more realistically the LP inhibition by the PD neurons. To analyze the response to periodic hyperpolarization, we only used the last 10 steps to avoid the transient responses of the LP neuron to the first few steps (Schneider et al., 2022).

### Data analysis

All data were imported from Clampex to Matlab (version 2021a) using the “abfload” function (Hentschke, 2011) and analyzed with custom-written scripts and the “CircStat” toolbox (Berens, 2009). Statistical tests were performed with SigmaPlot (version 12.0, Systat Software) with a significance level of *α* = 0.05. We used ANOVA with Tukey's post hoc test if the data passed tests for normal distribution (Shapiro–Wilk) and equal variance (Levene). If one of these tests failed, we used ANOVA on ranks with Dunn's post hoc test to compare the metrics between different modulatory conditions.

#### Pyloric rhythm attributes

To quantify the pyloric rhythm (Fig. 1*C*), we used extracellular nerve recordings to calculate the cycle period (*P *= time difference between the onsets of two consecutive PD bursts), and, for PD, LP, and pyloric constrictor (PY) each, the latencies of burst onset (e.g., LP_on_) and termination (e.g., LP_off_) relative to PD burst onset, number of spikes per burst (#spks), and mean intraburst spike frequency (*F*_spks_). Cycle frequency (*F*_cycle_) was calculated as 1 / *P*, and the burst onset and end phases were calculated as the respective latency divided by cycle period. For example, the end phase of the LP burst is given by ϕLP_off _= LP_off_ / *P*. Note that in each animal, PD exists in two copies and PY in 3–5 copies. Since we obtained #spks and *F*_spks_ from extracellular recordings, the values we report were from all active PD or PY units, detected on pdn and pyn, respectively. It is worth noting that we used bursting activity phases, rather than latencies, as important attributes to measure. The reason for this is that burst latencies, but not phases, show a strong correlation with *F*_cycle_ as has been noted in a number of publications (Bucher et al., 2005; Goaillard et al., 2009; Anwar et al., 2022; Cronin et al., 2024). Similarly, we used both *#*spks and *F*_spks_ because they are only weakly correlated in the pyloric circuit (Bucher et al., 2005; Cronin et al., 2024).

A complete quantification of the pyloric circuit activity requires the existence of a triphasic rhythm. Some decentralized, unmodulated preparations do not express rhythmic activity and were treated as missing values in the analysis, indicated as “NaN” in the raw data tables (see Extended Data). We did not remove any outliers from our datasets in order to not constrain interindividual variability. Criteria to exclude experiments were no robust triphasic rhythm in the intact condition or input resistances lower than 5 MΩ in the LP neuron.

We used different metrics to measure the interindividual variability of the different attributes. For *F*_cycle_, *#*spks, and *F*_spks_, we used the adjusted coefficient of variation (CV), which is the standard deviation divided by the mean. As recommended for small sample sizes, we also made a bias adjustment for the value of CV by a multiplicative factor of 1 + 1 / (4*n*), where *n* is the sample size (Haldane, 1955; Schillaci and Schillaci, 2022). Since CV is meaningful only for data on a ratio scale, we used the circular variance as a measure of variability for burst phase (*ϕ*). Briefly, each value of *ϕ* is represented as a vector composed of the sine and cosine of its angle on a unit circle with a length of 1. Averaging these vectors gives a resultant vector with the mean *ϕ* as direction and a length (*r*) between 0 and 1 that depends on the spread of the data. If all values of *ϕ* data were identical and therefore located at the same point on the unit circle, the *r*-vector would have a length of 1. If all data were evenly spread around the circle, the *r*-vector length would be 0. Circular variance is, by definition, 1 − *r*. Since the circuit neurons do not have independent activity phases, we computed the correlation coefficient matrices for all phases for each modulatory condition and their eigenvalues. We then summed the eigenvalues, which yields a measure for the variability of all phase data. Similarly, we summed the eigenvalues for all pooled *#*spks and *F*_spks_.

#### Single-neuron excitability attributes

We analyzed the current-clamp data as described in Schneider et al., (2022). In brief, we fitted the *f–I* curves with the following power function:

$$f(I)=a(I-I_{0}{)}^{b},\;f\geq 0,$$

where *a* is a scaling factor, *I*_0_ is the (calculated) current level that first elicited spikes, and the power *b* (set to 0 ≤ *b *≤ 1 to limit the *f–I* curve to a sublinear function) measures the nonlinearity (curvature) of the *f–I* curve. Hysteresis was calculated as the ratio of the average spike frequency for injected current between 2 and 4 nA with increasing current steps divided by the average spike frequency between 2 and 4 nA with decreasing current steps.

For the rebound protocols, we considered all five sweeps for the 10 s current step and only the last 10 sweeps for the 1 s current step. We measured the average latency between the end of the current step and the first spike. We fitted the cumulative spike histogram with the sigmoid as follows:

$$f(t)=\frac{a}{1+\exp\;((t-t_{1/2})/k)},$$

where *a* is the total number of spikes in the interval between current steps, *t*_1/2_ is the sigmoid midpoint time relative to the end of current step, and *k* is the slope factor.

As a metric for interindividual variability of fit parameter, hysteresis, and latency, we used either the adjusted CV or standard deviation for interval measures (for which CV was not applicable).

## Results

### Comodulation at mid concentrations reduced interindividual variability of the circuit activity attributes

The intact stomatogastric nervous system consists of the stomatogastric ganglion (STG), which includes the pyloric circuit neurons, as well as the esophageal ganglion (OG) and the paired commissural ganglia (CoGs; Fig. 1*A*). A variety of small-molecule neurotransmitters and neuropeptides modulate circuit activity in the STG, either as neurohormones present in the hemolymph or released from descending projection neurons. These projection neurons, many of which are spontaneously active at any time, have their cell bodies in the OG and CoGs and send axons to the STG via the stomatogastric nerve (stn). We characterized the pyloric rhythm by determining output pattern attributes from simultaneous extracellular recordings of the three motor nerves, lvn, pdn, and pyn (Fig. 1*A*). The core circuit that generates the pyloric rhythm (Fig. 1*B*) includes the anterior burster (AB) interneuron as well as two pyloric dilator (PD), lateral pyloric (LP), and 3–5 pyloric constrictor (PY) motor neurons. The axons of the three motor neuron types, PD, LP, and PY, project bilaterally through the lvn, and their action potentials can be readily separated by simultaneous extracellular recordings of lvn, pdn, and pyn that show the well-characterized triphasic pyloric burst sequence of these neurons (Fig. 1*C*; Marder and Bucher, 2007).

**Figure 2.**
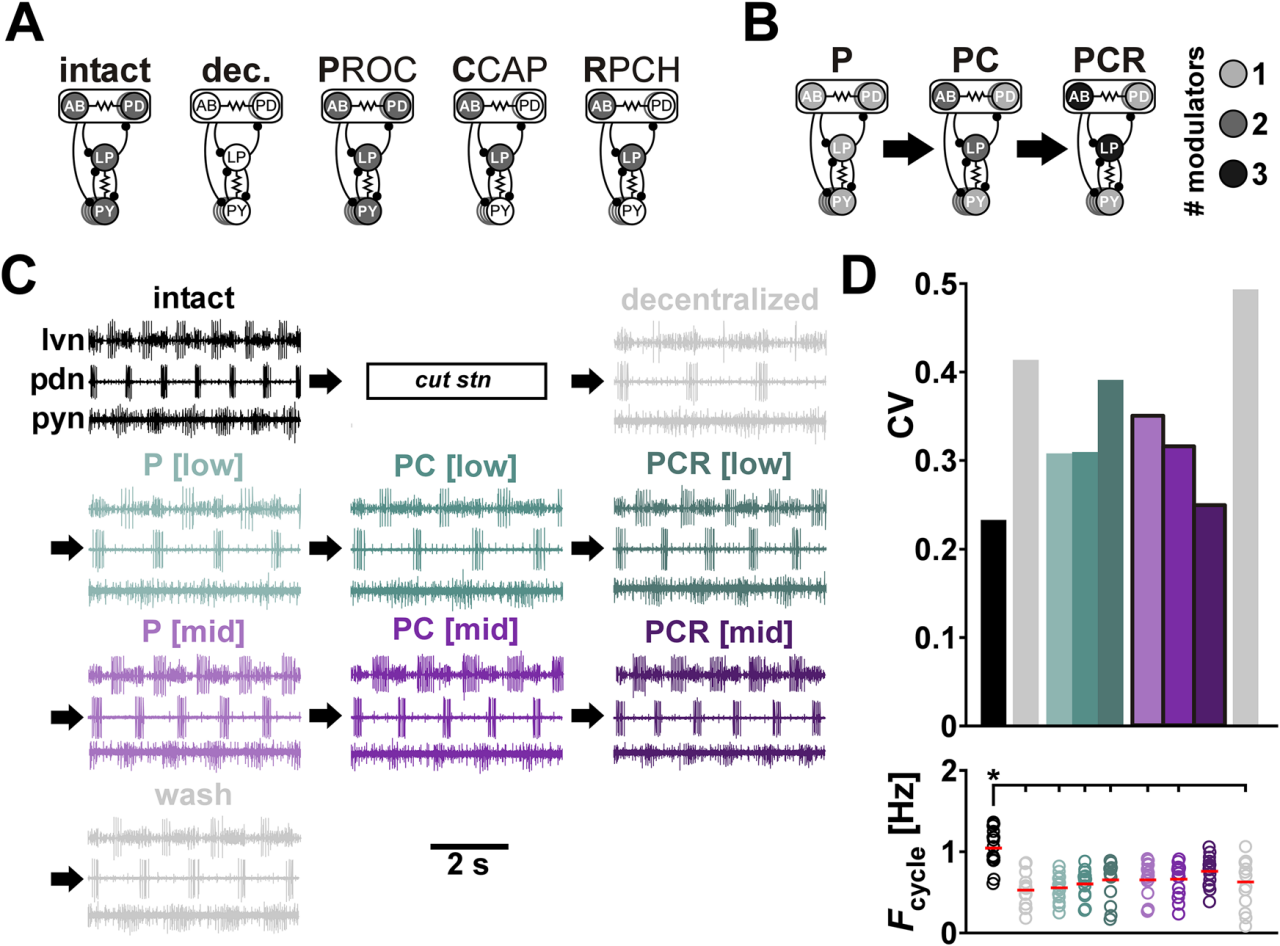
Comodulation at mid concentrations reduces the interindividual variability of cycle period. ***A***, Schematic indication of neuromodulator target neurons (filled circles) in the different neuromodulatory conditions. In the intact condition, each neuron is targeted by an unknown number of neuromodulators. After decentralization (transection of the stomatogastric nerve), all neuromodulation is removed. Proctolin (PROC, P) targets all pyloric neurons, whereas CCAP (C) and RPCH (R) target only AB and LP neurons. ***B***, Overlapping targets of comodulation by PROC, CCAP, and RPCH. Shading indicates how many of the applied neuromodulators target each neuron of the pyloric circuit. ***C***, Extracellular recordings of the pyloric rhythm from one animal under different neuromodulatory conditions (color coded). After decentralization (dec.), we applied increasing numbers (P, PC, PCR) and increasing concentrations ([low]: 10^−9^ M, [mid]: 3 × 10^−8^ M) of neuropeptides (arrows), followed by washing out all neuromodulators. ***D***, *F*_cycle_ and the corresponding CV (standard deviation/mean) under different modulatory conditions. Bottom panel: Individual dots represent data from individual experiments; red bars indicate the mean value. *N* = 15 animals. The asterisk indicates pairwise significant differences between the group indicated with the longer line and those indicated with shorter lines. All other pairwise comparisons were not statistically significant. Statistical results are shown in Table 1. Total modulator concentration for [low]: 10^−9^ M; [mid]: 3 × 10^−8^ M.

We measured pyloric output pattern attributes (marked in Fig. 1*C*) across different modulatory states, with intact descending input, after decentralization (see below), and after application of neuropeptides (Fig. 2*A*). With intact descending inputs, spontaneous activity of projection neurons ensures that the pyloric neurons are always bathed in a mixture of modulatory neurotransmitters, including several peptides that are coreleased by these projection neurons (Nusbaum et al., 2017). In vitro, all modulatory inputs can be removed by blocking or severing the projection nerve (stn), a procedure we refer to as decentralization (Fig. 2*A*). Removing all neuromodulatory input to the STG by decentralization greatly slows or even disrupts the pyloric rhythm (Hamood and Marder, 2015). Bath application of excitatory neuropeptides produces a peptide-specific version of the pyloric rhythm. For example, CCAP significantly increases the firing rate of the LP neuron, whereas PROC elicits a rhythm similar to that seen in the intact system (Marder and Weimann, 1992; Swensen and Marder, 2001; Marder and Thirumalai, 2002; but see Cronin et al., 2024). We used three excitatory neuropeptides, PROC, CCAP, and RPCH, that target distinct but overlapping subsets of neurons in the pyloric circuit (Fig. 2*A*), but that all converge on the same target ion channel within these subsets. This target is a voltage-gated inward current referred to as *I*_MI_ (Golowasch and Marder, 1992; Swensen and Marder, 2000; Gray and Golowasch, 2016; Gray et al., 2017; Schneider et al., 2021). PROC activates *I*_MI_ in AB, PD, LP, and most PY neurons, while both CCAP and RPCH activate *I*_MI_ only in AB and LP neurons (Fig. 2*A*; Swensen and Marder, 2001). Additionally, both PROC and CCAP enhance synaptic currents in the reciprocal connections between the pacemaker neurons and LP (Li et al., 2018), but their modulatory effects on the pacemaker to PY and PY to LP synapses have not been characterized. RPCH increases the strength of the LP to pacemaker synapse (Thirumalai et al., 2006; Atamturktur and Nadim, 2011), but its effects on other synapses have not been studied.

Our goal was to test whether comodulation by multiple excitatory neuropeptides reduces interindividual variability of circuit output. To examine this hypothesis, we bath-applied combinations of one (PROC = P), two (PROC + CCAP = PC), and three (PROC + CCAP + RPCH = PCR) neuropeptides at the same total low and mid concentrations (Fig. 2*B*,*C*) in several preparations and examined the variability of multiple circuit output attributes (see Materials and Methods for detailed description). Pyloric circuit output attributes of interest (Fig. 1*C*) fall into different categories that are qualitatively distinct and typically also have distinct units. We therefore divided these attributes into three general categories [cycle frequency (*F*_cycle_), neuronal burst phases, and the intraburst spiking activity of individual neuron types] and analyzed the effect of modulators on the variability of these components separately.

Upon decentralization, the *F*_cycle_ coefficient of variation (CV) approximately doubled compared with the intact state and remained high when we applied one, two, or three neuropeptides at low concentration (Fig. 2*D*, top panel; all detailed data for Fig. 2*D* are provided in Extended Data Fig. 2-1 and ANOVA results in Table 1). However, when we applied increasing numbers of peptides at mid concentration, the CV consistently decreased toward its intact value. Washing out the modulators increased CV as expected. Decentralization decreased the mean value of *F*_cycle_ compared with intact preparations (Fig. 2*D*). While application of one or more neuropeptides at low or mid concentrations to the decentralized preparation tended to increase *F*_cycle_, it was only with application of three modulators at mid concentration that *F*_cycle_ recovered to values statistically similar to those in the intact condition (Fig. 2*D*, bottom panel and Extended Data Fig. 2-1).

10.1523/ENEURO.0167-24.2024.f2-1 Figure 2-1 All data for the analysis shown in Figure 2D. The first column is the unique identifier for each experiment. A, B denotes the two preparations in the same dish from which we recorded simultaneously. Data columns show the average *F*_cycle_ in Hz over 30  s recordings (at least 10 bursts) for each experiment. Column headers indicate the experimental conditions in the order of application: intact, untreated decentralized (neuromodulatory inputs removed), low (10^−9^ M) concentrations of PROC (P [low]), PROC + CCAP (PC [low]), PROC + CCAP + RPCH (PCR [low]), mid (3 × 10^−8^ M) concentrations of the same neuropeptide combinations (P [mid], PC [mid], PCR [mid]), wash. NaN indicates that the preparation was not rhythmic in that condition. Download Figure 2-1, XLS file.

**Table 1.** ANOVA results for pyloric rhythm parameters at low and mid modulator concentrations

*N*	Parameter	Normality	Variance	Test statistic	df	res.	*p*
15	*F*_cycle_	Fail		*H* = 36.645	8		**<0**.**001**
15	PD_off_	Fail		*H* = 24.794	8		**0**.**005**
15	LP_on_	Fail		*H* = 19.799	8		**0**.**011**
15	LP_off_	Fail		*H* = 37.006	8		**<0**.**001**
15	PY_on_	Fail		*H* = 20.498	8		**0**.**009**
15	PY_off_	Fail		*H* = 15.495	8		0.050
15	PD #spks	Pass	Pass	*F* = 3.365	8	119	**0**.**002**
15	PD *F*_spks_	Fail		*H* = 29.773	8		**<0**.**001**
14	LP #spks	Pass	Pass	*F* = 21.217	8	111	**<0**.**001**
14	LP *F*_spks_	Pass	Fail	*H* = 50.238	8		**<0**.**001**
15	PY #spks	Fail		*H* = 9.413	8		0.309
15	PY *F*_spks_	Fail		*H* = 9.958	8		0.268

**Figure 3.**
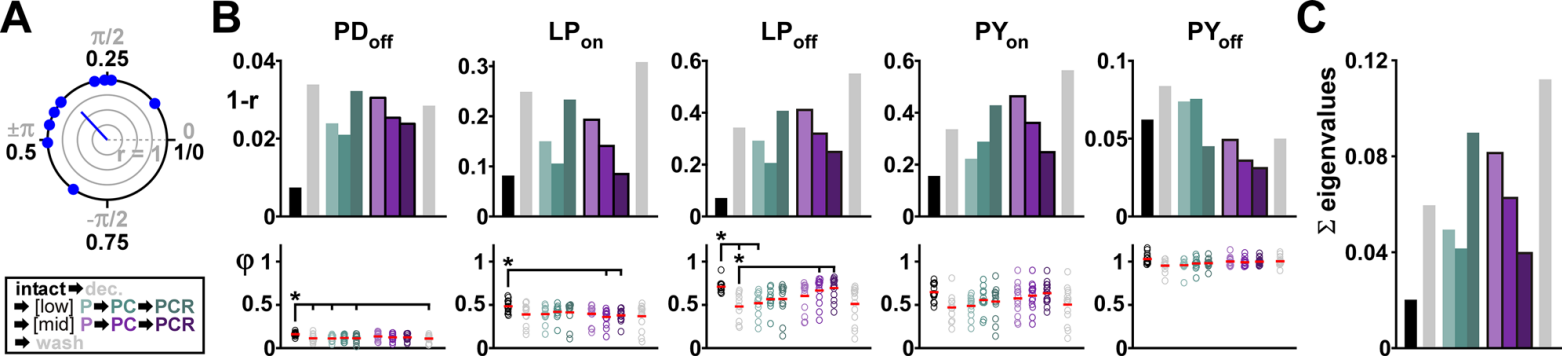
Comodulation at mid concentrations reduces the interindividual variability of pyloric rhythm parameters on the circuit output level. ***A***, Circular plot of a randomly generated phase dataset (*N* = 10; $\bar{x}$ = 0.35; *µ* = 0.15). The length of the *r*-vector indicates the spread of the data. We use 1 − *r* as measure for variability of circular data. Markers inside the circle indicate 25, 50, and 75% of the radius of the unit circle. ***B***, Burst start (on) and termination (off) and the corresponding circular variance (1 − *r*; see Materials and Methods) under different modulatory conditions (color coded). Individual dots represent data from individual experiments; red bars indicate the circular mean. Asterisks indicate pairwise significant differences between the group indicated with the longer line and those indicated with shorter lines. All other pairwise comparisons were not statistically significant. *N* = 15 animals. Statistical results are shown in Table 1. Total modulator concentration for [low]: 10^−9^ M; [mid]: 3 × 10^−8^ M. ***C***, Sum of eigenvalues from the covariance matrix of the phases shown in panel ***B*** for each modulatory condition.

If tests for normality or equal variance failed, results are for ANOVA on ranks. Groups are intact, decentralized, P [low], PC [low], PCR [low], P [mid], PC [mid], PCR [mid], wash. *p* values smaller than *α* are printed in bold. *N*, number of animals; df, degrees of freedom; res, residual. Note that despite the significant effect from ANOVA, the pairwise comparison for PY on phase did not show any significant differences between groups (Fig. 3*B*).

The burst onset (on) and end (off) phases of individual pyloric neurons within each cycle are a major determinant of the proper function of this central pattern generator, and these phases remain surprisingly consistent across individual animals despite large variations in *F*_cycle_ (Bucher et al., 2005; Goaillard et al., 2009; Anwar et al., 2022; Cronin et al., 2024). We measured variability of activity phases by calculating the circular variance (1 − *r*; Fig. 3*A*; also see Materials and Methods). Decentralization resulted in a general increase of circular variance of both on and off phases of all neurons (Fig. 3*B*, top panels) and application of neuropeptides, even at low concentrations, typically reduced the circular variance. Notably, for all neuronal burst onset and end phases, circular variance consistently decreased when we increased the number of applied neuropeptides at mid concentration but not at low concentration (Fig. 3*B*, top panels). Decentralization and application of neuropeptides affected the mean phases of PD and LP, but not of PY (Fig. 3*B*, bottom panels; detailed data for Fig. 3*B*,*C* are provided in Extended Data Fig. 3-1 and ANOVA results in Table 1). Because of the extensive connectivity of the pyloric circuit (Fig. 1*B*), the PD, LP, and PY on and off phases are not independent of each other. To account for the covariation of the phases, we computed the covariance matrix of all five phases in each modulatory condition. Total variation can be measured as the trace (equivalently, sum of the eigenvalues) of this matrix (Fig. 3*C*). The resulting pattern of total variation of phase under the different modulatory conditions was the same as for *F*_cycle_ and for individual phases: Variability increased with decentralization but consistently decreased when we increased the number of neuropeptides at mid concentrations. No consistent pattern was seen at low concentrations.

10.1523/ENEURO.0167-24.2024.f3-1 Figure 3-1 All data for the analysis shown in Figure 3B, C. The Excel file contains one sheet for each neuron type and phase (PD_off_, LP_on_, LP_off_, PY_on_, PY_off_). The first column is the unique identifier for each experiment. A, B denotes the two preparations in the same dish from which we recorded simultaneously. Data columns show the average phase on and phase off over 30  s recordings (at least 10 bursts) for each experiment. Column headers indicate the experimental conditions in the order of application: intact, decentralized (neuromodulatory inputs removed), low (10^−9^ M) concentrations of PROC (P [low]), PROC + CCAP (PC [low]), PROC + CCAP + RPCH (PCR [low]), mid (3 × 10^−8^ M) concentrations of the same neuropeptide combinations (P [mid], PC [mid], PCR [mid]), wash. NaN indicates that the respective neuron was not rhythmic in that condition. Download Figure 3-1, XLS file.

One caveat of these experiments was that, because we had applied the neuropeptides sequentially at low and then mid concentrations (Fig. 2*C*), the preparations recorded with mid concentration of P, PC, and PCR had all been pre-exposed to low concentrations of all three modulators (low PCR). To ensure that the effects of comodulation seen at mid concentration were not due to history dependence, we repeated these experiments by applying P, PC, and PCR at only mid concentrations. These experiments effectively replicated the result that most attributes of the pyloric circuit output showed a reduction of variability with increased numbers of peptide comodulators (Extended Data Fig. 3-2; detailed data are provided in Extended Data Fig. 3-3 and ANOVA results in Table 2).

10.1523/ENEURO.0167-24.2024.f3-2 Figure 3-2 Comodulation at mid concentrations reduces the interindividual variability of rhythm parameters on the circuit output level in an independent dataset. **(A)** *F*_cycle_ and the corresponding CV under different modulatory conditions. **(B)** Burst start (on) and termination (off) and the corresponding circular variance, 1 - *r*, see Methods) under different modulatory conditions (color coded). N = 25 animals. Individual dots represent data from individual experiments, red bars indicate the (circular) mean. Asterisks indicate pairwise significant differences between two groups, or the group indicated with the longer line and those indicated with shorter lines (Dunn’s post-hoc test, p ≤ 0.05). The groups indicated with daggers are significantly different from all other groups but not from one another in that panel. All other pairwise comparisons were not statistically significant. Statistical results in Table 2. Download Figure 3-2, TIF file.

10.1523/ENEURO.0167-24.2024.f3-3 Figure 3-3 All data for the analysis shown in Figure 3-3. The Excel file contains one sheet for each F_cycle_, and neuron type and phase (PD_off_, LP_on_, LP_off_, PY_on_, PY_off_). The first column is the unique identifier for each experiment. A, B denotes the two preparations in the same dish from which we recorded simultaneously. Data columns show the average phase on and phase off over 30  s recordings (at least 10 bursts) for each experiment. Column headers indicate the experimental conditions in the order of application: intact, decentralized (neuromodulatory inputs removed), low (10^−9^ M) concentrations of PROC (P [low]), PROC + CCAP (PC [low]), PROC + CCAP + RPCH (PCR [low]), mid (3 × 10^−8^ M) concentrations of the same neuropeptide combinations (P [mid], PC [mid], PCR [mid]), wash. NaN indicates that the respective neuron was not rhythmic in that condition. Starting from column I are the results of the 1-way ANOVA on ranks and multiple comparisons. Download Figure 3-3, XLS file.

**Table 2.** ANOVA results for pyloric rhythm attributes at mid modulator concentrations (different dataset than in Table 1)

*N*	Attribute	Normality	Variance	Test statistic	df	res.	*p*
25	*F*_cycle_	Fail		*H* = 61.109	5		**<0**.**001**
25	PD_off_	Fail		*H* = 33.152	5		**<0**.**001**
25	LP_on_	Pass	Fail	*H* = 37.630	5		**<0**.**001**
25	LP_off_	Pass	Fail	*H* = 61.122	5		**<0**.**001**
25	PY_on_	Pass	Pass	*F* = 7.677	5	134	**<0**.**001**
25	PY_off_	Pass	Pass	*F* = 7.224	5	134	**<0**.**001**
25	PD #spks	Fail		*H* = 31.084	5		**<0**.**001**
25	PD *F*_spks_	Fail		*H* = 5.576	5		0.350
25	LP #spks	Pass	Fail	*H* = 94.633	5		**<0**.**001**
25	LP *F*_spks_	Pass	Pass	*F* = 29.448	5	131	**<0**.**001**
25	PY #spks	Fail		*H* = 41.5592	5		**<0**.**001**
25	PY *F*_spks_	Pass	Pass	*F* = 3.009	5	134	**0**.**013**

If tests for normality or equal variance failed, results are for ANOVA on ranks. Groups are intact, decentralized, P [mid], PC [mid], PCR [mid], wash. *p* values smaller than *α* are printed in bold. *N*, number of animals; df, degrees of freedom; res, residual.

**Figure 4.**
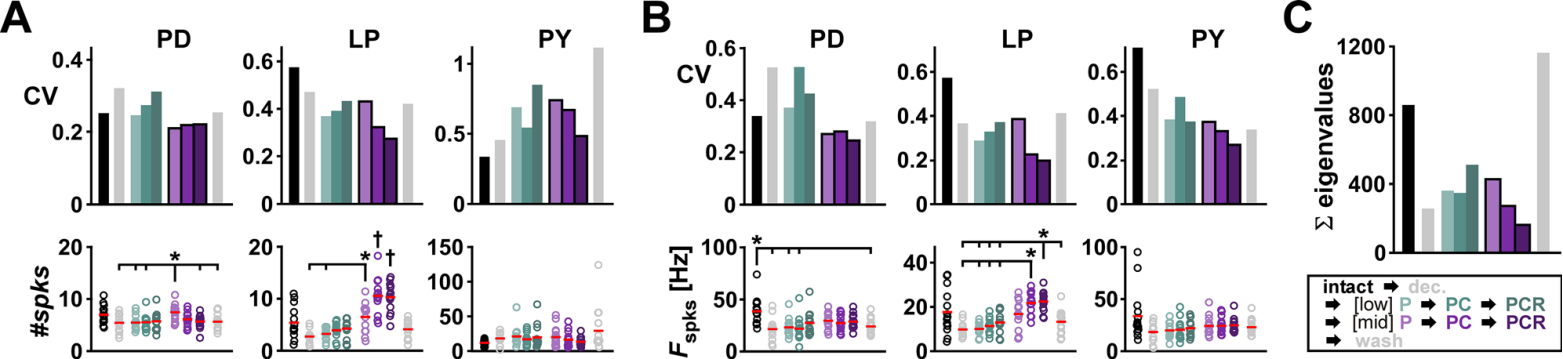
Comodulation at mid concentrations reduces the interindividual variability of burst parameters on the circuit output level. ***A***, Average number of spikes (#spks) per burst and corresponding CV for each type of neuron at each modulatory condition (color coded). ***B***, Average spike frequency (*F*_spks_) within a burst and corresponding CV for each type of neuron at each modulatory condition (color coded). ***C***, Sum of eigenvalues from the covariance matrix of pooled #spks and *F*_spks_ for each modulatory condition. Asterisks indicate pairwise significant differences between the group indicated with the longer line and those indicated with shorter lines. The two groups indicated with daggers in panel ***A*** are significantly different from all other groups but not one another. All other pairwise comparisons were not statistically significant. *N* = 15 animals. Dots represent values from individual experiments; horizontal bars indicate the mean values. Statistical results in Table 1. Total modulator concentration for [low]: 10^−9^ M; [mid]: 3 × 10^−8^ M.

Finally, we also quantified two other important output attributes of the pyloric circuit neurons: #spks and *F*_spks_. In PD, the mean #spks was largely unaffected by decentralization and modulator application (Fig. 4*A*, bottom left), but PD *F*_spks_ reduced with decentralization and only mid-concentration peptide application returned this attribute toward its intact values (Fig. 4*B*, bottom left). LP had a significantly larger #spks and *F*_spks_ in PC and PCR at mid concentrations (Fig. 4*A*,*B*, bottom middle). In contrast, mean spiking activity of PY remained unchanged across all conditions (detailed data are provided in Extended Data Fig. 4-1, ANOVA results in Table 1). The changes in interindividual variability of the spiking activity (measured as CVs) were somewhat different from the consistent changes we saw for *F*_cycle_ and the activity phases. For these attributes, in both the LP and PY neurons, the CVs of both spiking attributes were reduced by adding modulators at mid concentration but not low concentration (Fig. 4*A*,*B*, top middle and right). However, the CVs of PD #spks and *F*_spks_ did not consistently decrease with adding modulators at mid concentration but remained similar across those conditions. In the experiments without prior exposure to low modulator concentration, once again, the CVs of PD spike attributes did not decrease with comodulation. In these experiments, we also did not see a consistent reduction in variability of PY spike attributes in the transition from two (PC) to three (PCR) modulators (Extended Data Fig. 4-2; detailed data are provided in Fig. 4-3 and ANOVA results in Table 2).

10.1523/ENEURO.0167-24.2024.f4-1 Figure 4-1 All data for the analysis shown in Figure 4. The Excel file contains one sheet for each neuron type and rhythm parameter (PD_#spks_, LP_#spks_, PY_#spks_, PD_F_spks_ LP_F_spks_, PY_F_spks_). The first column is the unique identifier for each experiment. A, B denotes the two preparations in the same dish from which we recorded simultaneously. Data columns show the average number of spikes (#spks) and spike frequency (F_spks_) in Hz within a burst over 30  s recordings (at least 10 bursts) for each experiment. Column headers indicate the experimental conditions in the order of application: intact, decentralized (neuromodulatory inputs removed), low (10^−9^ M) concentrations of PROC (P [low]), PROC + CCAP (PC [low]), PROC + CCAP + RPCH (PCR [low]), mid (3 × 10^−8^ M) concentrations of the same neuropeptide combinations (P [mid], PC [mid], PCR [mid]), wash. NaN indicates that the respective neuron was not active or did not generate enough spikes to calculate spike frequency in that condition. Download Figure 4-1, XLS file.

10.1523/ENEURO.0167-24.2024.f4-2 Figure 4-2 Comodulation at mid concentrations reduces the interindividual variability of rhythm parameters on the circuit output level in an independent dataset. **(A)** Average number of spikes (#spks) per burst and corresponding CV for each type of neuron at each modulatory condition (color coded). **(B)** Average spike frequency (*F*_spks_) within a burst and corresponding CV for each type of neuron at each modulatory condition (color coded). N = 25 animals. Individual dots represent data from individual experiments, red bars indicate the (circular) mean. Asterisks indicate pairwise significant differences between two groups, or the group indicated with the longer line and those indicated with shorter lines (Dunn’s post-hoc test, p ≤ 0.05). The groups indicated with daggers are significantly different from all other groups in that panel. All other pairwise comparisons were not statistically significant. Statistical results in Table 2. Download Figure 4-2, TIF file.

10.1523/ENEURO.0167-24.2024.f4-3 Figure 4-3 All data for the analysis shown in Figure 4-3. The Excel file contains one sheet for each neuron type and rhythm parameter (PD_#spks_, LP_#spks_, PY_#spks_, PD_F_spks_ LP_F_spks_, PY_F_spks_). The first column is the unique identifier for each experiment. A, B denotes the two preparations in the same dish from which we recorded simultaneously. Data columns show the average phase on and phase off over 30  s recordings (at least 10 bursts) for each experiment. Column headers indicate the experimental conditions in the order of application: intact, decentralized (neuromodulatory inputs removed), low (10^−9^ M) concentrations of PROC (P [low]), PROC + CCAP (PC [low]), PROC + CCAP + RPCH (PCR [low]), mid (3 × 10^−8^ M) concentrations of the same neuropeptide combinations (P [mid], PC [mid], PCR [mid]), wash. NaN indicates that the respective neuron was not rhythmic in that condition. Starting from column I are the results of the 1-way ANOVA and multiple comparisons. Download Figure 4-3, XLS file.

As with the case of the pyloric phases, the spiking activities of the bursting pyloric neurons are not independent of each other. To account for the covariation of the spike frequency and number of spikes, we computed the covariance matrix of #spks and *F*_spks_ in each modulatory condition. The sum of the eigenvalues of this matrix describes the total variation of these outputs (Fig. 4*C*). As with *F*_cycle_ and the phases, total spiking variability increased with decentralization but consistently decreased when we increased the number of neuropeptides at mid concentrations. No consistent pattern was seen at low concentrations.

Cycle-to-cycle variability of the pyloric rhythm within animals is known to be much lower than variability across animals in intact conditions but decentralization increases cycle-to-cycle variability within an animal (Bucher et al., 2005; Hamood et al., 2015). We examined the effect of peptide comodulation at low and mid concentrations on the cycle-to-cycle variability. In contrast to the results across animals, we did not find comodulation by increasing numbers of neuropeptides to consistently reduce cycle-to-cycle variability within animals (Table 3).

**Table 3.** Cycle-by-cycle variability within each animal averaged across experiments (± standard deviation) at different modulatory conditions

	intact	dec.	[low] P	[low] PC	[low] PCR	[mid] P	[mid] PC	[mid] PCR	wash
Variability *F*_cycle_	0.023 ± 0.010	0.085 ± 0.010	0.042 ± 0.011	0.058 ± 0.011	0.059 ± 0.011	0.026 ± 0.011	0.058 ± 0.012	0.042 ± 0.009	0.027 ± 0.009
Variability PD_off_	0.006 ± 0.006	0.007 ± 0.006	0.006 ± 0.006	0.006 ± 0.006	0.005 ± 0.006	0.003 ± 0.006	0.005 ± 0.012	0.008 ± 0.012	0.008 ± 0.012
Variability LP_on_	0.008 ± 0.010	0.030 ± 0.010	0.011 ± 0.010	0.006 ± 0.010	0.005 ± 0.010	0.002 ± 0.010	0.014 ± 0.010	0.015 ± 0.010	0.003 ± 0.010
Variability LP_off_	0.050 ± 0.107	0.049 ± 0.017	0.022 ± 0.017	0.020 ± 0.017	0.013 ± 0.017	0.011 ± 0.017	0.015 ± 0.018	0.025 ± 0.018	0.010 ± 0.013
Variability PY_on_	0.028 ± 0.024	0.050 ± 0.020	0.043 ± 0.020	0.044 ± 0.020	0.039 ± 0.021	0.023 ± 0.022	0.033 ± 0.021	0.040 ± 0.020	0.026 ± 0.026
Variability PY_off_	0.053 ± 0.050	0.045 ± 0.047	0.043 ± 0.047	0.032 ± 0.047	0.032 ± 0.047	0.028 ± 0.047	0.032 ± 0.048	0.047 ± 0.047	0.041 ± 0.047
Variability PD #spks	0.116 ± 0.051	0.134 ± 0.062	0.130 ± 0.053	0.118 ± 0.052	0.095 ± 0.049	0.090 ± 0.049	0.091 ± 0.050	0.112 ± 0.050	0.096 ± 0.049
Variability PD *F*_spks_	0.109 ± 0.053	0.120 ± 0.052	0.121 ± 0.052	0.117 ± 0.054	0.111 ± 0.052	0.089 ± 0.052	0.111 ± 0.051	0.111 ± 0.051	0.110 ± 0.051
Variability LP #spks	0.169 ± 0.116	0.234 ± 0.108	0.169 ± 0.104	0.118 ± 0.101	0.126 ± 0.103	0.104 ± 0.237	0.219 ± 0.240	0.091 ± 0.242	0.168 ± 0.242
Variability LP *F*_spks_	0.116 ± 0.074	0.154 ± 0.053	0.110 ± 0.051	0.091 ± 0.048	0.063 ± 0.051	0.069 ± 0.056	0.105 ± 0.057	0.097 ± 0.055	0.071 ± 0.050
Variability PY #spks	0.173 ± 0.079	0.258 ± 0.074	0.205 ± 0.070	0.195 ± 0.067	0.196 ± 0.066	0.164 ± 0.069	0.230 ± 0.067	0.211 ± 0.067	0.193 ± 0.066
Variability PY *F*_spks_	0.157 ± 0.061	0.109 ± 0.066	0.112 ± 0.061	0.114 ± 0.060	0.126 ± 0.060	0.134 ± 0.060	0.150 ± 0.059	0.153 ± 0.060	0.114 ± 0.059

Variability for phases was measured as circular variance, and as CV for all other attributes. Same dataset as in Table 1.

### Compared with single peptide modulators, comodulation at mid concentrations did not reduce interindividual variability at the single-neuron level

A previous study has shown that neuropeptide modulation at saturating concentration reduces the interindividual variability of excitability attributes of the synaptically isolated LP neuron (Schneider et al., 2022). This finding raises the possibility that the reduction of circuit output variability arising by increasing numbers of comodulatory peptides at mid-level concentration may be a consequence of a similar reduction at the level of individual pyloric neurons. To examine this hypothesis, we measured single-neuron interindividual variability using the same protocols as in Schneider et al. (2022), but with increasing numbers of comodulators applied at mid concentration since we observed a reduction of variability on the network level only at that concentration. As in that previous study, we focused on the synaptically isolated LP neuron because it exists only in a single copy in each animal, and it is targeted by all three neuropeptides that we used in this study (Swensen and Marder, 2001).

**Figure 5.**
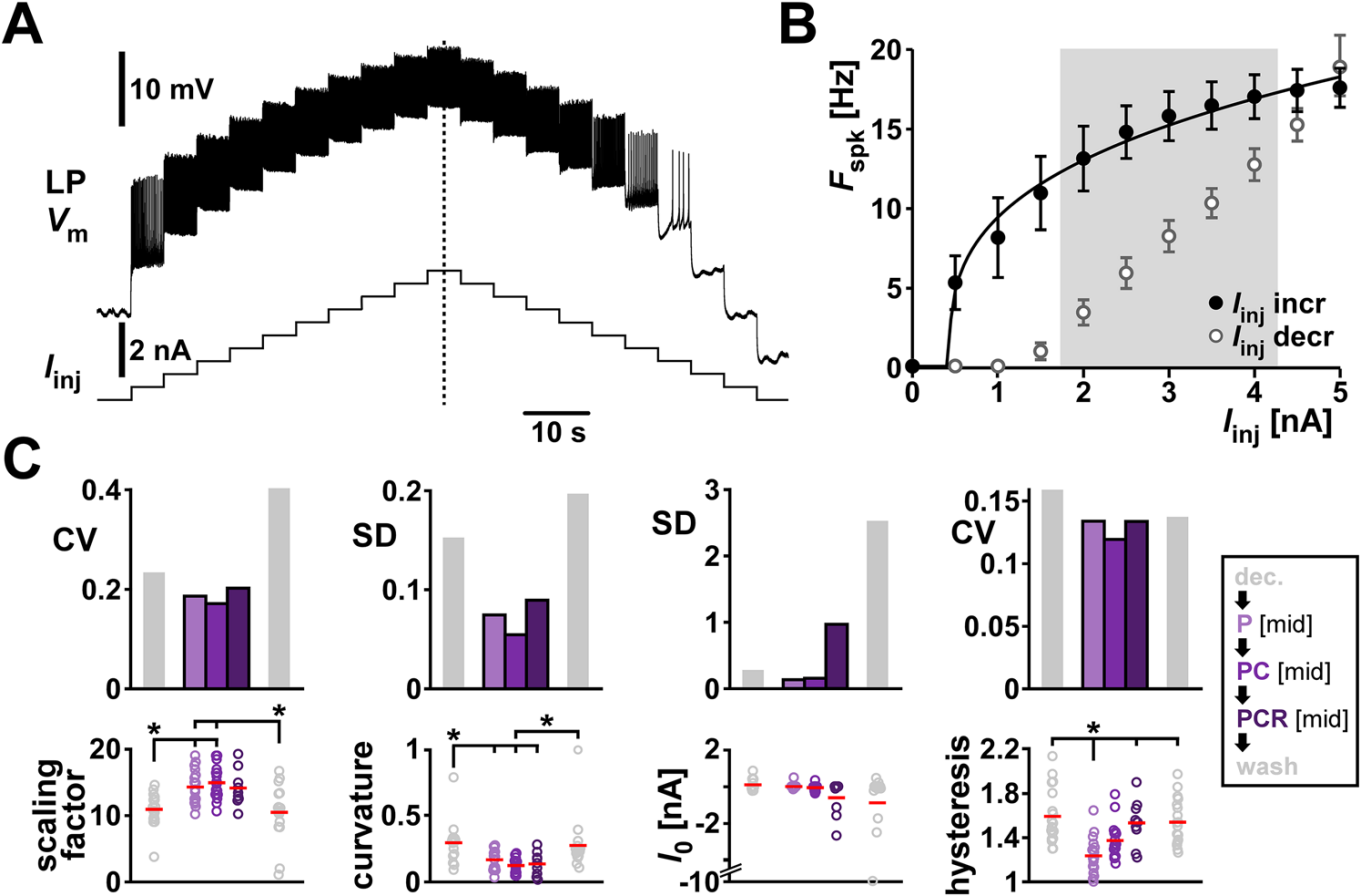
Comodulation does not reduce the interindividual variability of excitability metrics on the single cell level. ***A***, Voltage changes of one example LP neuron in response to increasing and decreasing current step. ***B***, *f–I* curve of the experiment in ***A***. The average spike frequency at each current level (dots and error bars indicate mean ± SD) was fitted with a power function (black line). To calculate *f–I* hysteresis between increasing (filled circles) and decreasing (open circles) levels of current steps, we divided the average spiking frequency in increasing by decreasing current levels between 2 and 4 nA (shaded area). ***C***, Fit parameters (scaling factor = *a*, curvature = *b* in Eq. 1) and hysteresis with the corresponding metric of variability (CV, or SD for interval data) for different modulatory conditions (color coded). Dots represent values from individual experiments; horizontal bars indicate the mean values. Asterisks indicate pairwise significant differences between two groups or the group indicated with the longer line and those indicated with shorter lines. All other pairwise comparisons were not statistically significant. *N* = 19, except PCR *N* = 11. Statistical results are shown in Table 4. Total modulator concentration for [mid]: 1–3 × 10^−8^ M for P and PC, 3 × 10^−8^ M for PCR.

The first excitability attribute that we examined was the spike frequency versus input current relationship (*f–I* curve). We measured the *f–I* curves by applying increasing and decreasing current steps and measured the firing frequency at each step (Fig. 5*A*). In order to compare these data across individuals, we parameterized the *f–I* curves across the range of applied currents by fitting these data with the sublinear power function provided in Equation 1 (Fig. 5*B* fit curve; also see Materials and Methods; Eq. 1). Both the scaling factor *a* and the exponent *b*, but not the zero intercept *I*_0_, were different in mean value in the presence of neuropeptides compared with the unmodulated condition, indicating a higher LP spike rate in the presence of the neuropeptides (detailed data are provided in Extended Data Fig. 5-1 and ANOVA results in Table 4). In general, fit parameter variability was lower when one or more peptide modulators were present, but there was no consistent reduction in variability with the addition of comodulators (Fig. 5*C*).

10.1523/ENEURO.0167-24.2024.f5-1 Figure 5-1 All data for the analysis shown in Figure 5C. The Excel file contains four sheets, one for each fit parameter (scaling factor, curvature, I0) and hysteresis. In each sheet the first column is the unique identifier for each experiment. Data columns show the values for each experiment. Column headers indicate the experimental conditions in the order of application: intact, decentralized (neuromodulatory inputs removed), mid (3 × 10^−8^ M) concentrations of PROC (P [mid]), PROC + CCAP (PC [mid]), PROC + CCAP + RPCH (PCR [mid]), wash. NaN indicates that the respective neuron or preparation was not active in that condition. Empty cells indicate that that neuromodulator combination was not applied to those experiments. Download Figure 5-1, XLS file.

**Table 4.** ANOVA (on ranks) results for *f*–*I* parameters

*N*	Parameter	Normality	Test statistic	df	*P*
17	Scaling factor (a)	Fail	*H* = 24.667	4	**<0.001**
17	Curvature (b)	Fail	*H* = 29.801	4	**<0**.**001**
17	*I*_0_	Fail	*H* = 3.680	4	0.451
19	Hysteresis	Fail	*H* = 27.796	4	**<0**.**001**

Groups are decentralized, P [mid], PC [mid], PCR [mid], wash. *p* values smaller than *α* are printed in bold. *N*, number of animals; df, degrees of freedom.

As seen in the voltage trace of Figure 5*A*, the *f–I* curve shows hysteresis, in that the firing frequency for each current step depends on whether it is measured on the increasing or decreasing leg of injected currents (Fig. 5*B*). We quantified hysteresis, as detailed in the Materials and Methods, by calculating the ratio between the mean *f* value on the increasing and decreasing legs of injected currents within the shaded region (2–4 nA) of Figure 5*B*. Thus, a value greater than 1 means that LP was spiking at a higher frequency when the current changed in increasing direction. Even though neuropeptides influenced hysteresis, they did not change its variability (Fig. 5*C*).

**Figure 6.**
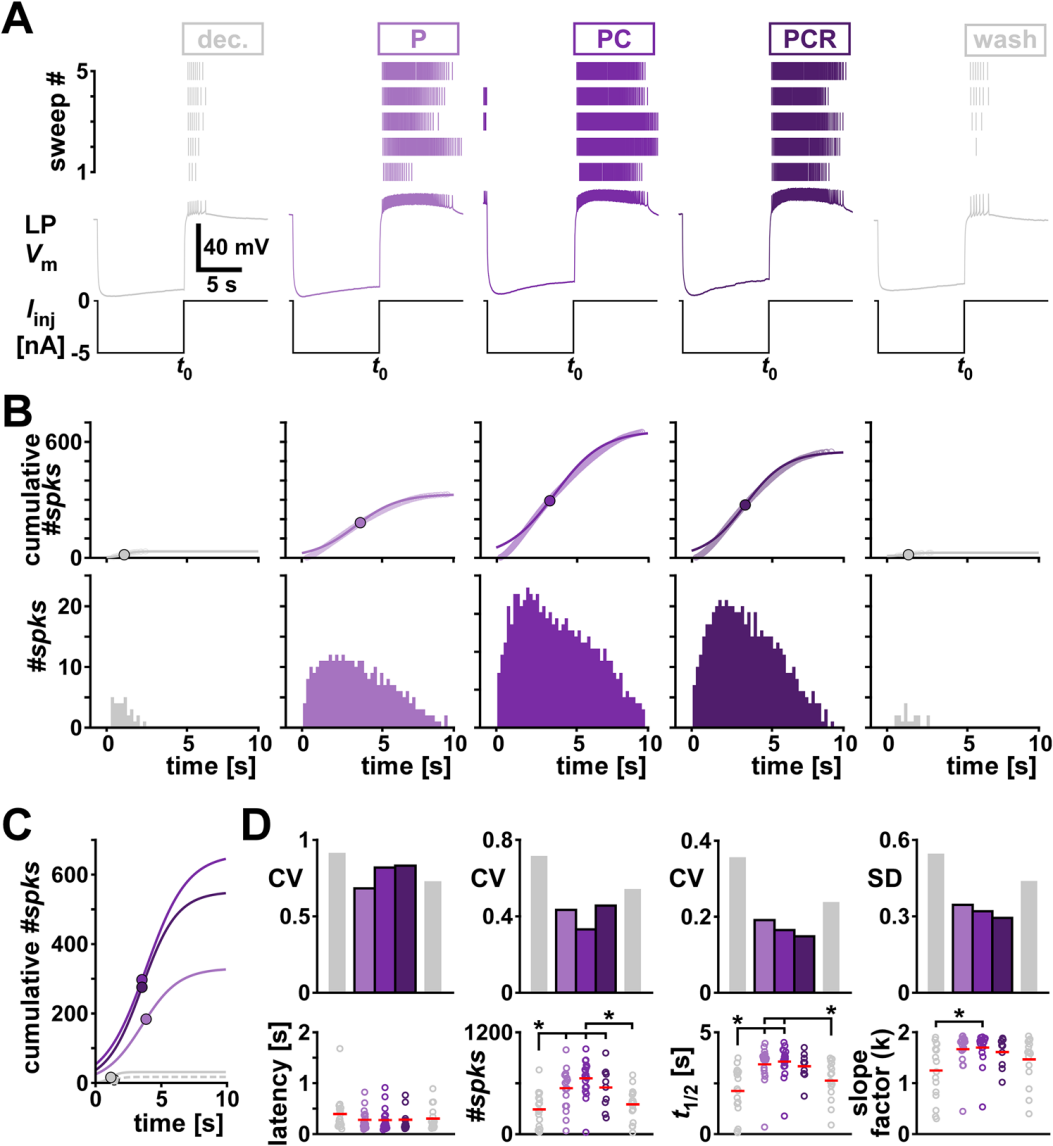
Comodulation does not add to the reduction of interindividual variability of rebound metrics on the single cell level. ***A***, An example of the rebound experiment in different modulatory conditions (color coded). The top panels show the spike raster plots for all five sweeps. The bottom panels show the intracellular voltage of one example sweep. *t*_0_ indicates the time point when LP was released from current injection. ***B***, Bottom row, Spike histograms across all sweeps in different modulatory conditions (color coded). Top row, Corresponding cumulative histograms of the panels in the bottom row (circles) and sigmoid fits to the cumulative spike histograms. Dots indicate the sigmoid midpoint. ***C***, Sigmoid fits to the cumulative spike histograms from panel B overlayed for comparison. Dots indicate the sigmoid midpoint. Dashed gray line indicates wash. ***D***, Latency and fit parameters (#*spks* = a, slope factor = *k* in Eq. 2) with the corresponding metric of variability (CV, or SD for interval data) for different modulatory conditions (color coded). Dots represent values from individual experiments; horizontal bars indicate the mean values. Asterisks indicate pairwise significant differences between two groups, or the group indicated with the longer line and those indicated with shorter lines. All other pairwise comparisons were not statistically significant. *N* = 19, except PCR *N* = 11. Statistical results are shown in Table 5. Total modulator concentration for [mid]: 1–3 × 10^−8^ M for P and PC, 3 × 10^−8^ M for PCR.

LP is a follower neuron and, during normal pyloric activity, generates bursts of spikes upon rebound from inhibition by the pacemaker neurons AB and PD. To examine the postinhibitory rebound properties of LP, we hyperpolarized the isolated LP neuron for 10 s and characterized the spiking activity in the 10 s window after release from inhibition in different modulatory conditions (Fig. 6*A*). We did so by measuring the latency from the end of inhibition to the first spike and generated spike histograms from the 10 s window following the inhibition (Fig. 6*B*). We characterized the spike histogram by fitting a sigmoid function shown in Equation 2 to the cumulative spike count (Fig. 6*B*, top row; overlayed in Fig. 6*C*).

Adding one or several neuropeptides at mid concentration significantly changed the mean values of the fit parameters of the sigmoid but not the rebound latency (Fig. 6*D*, bottom row; detailed data are provided in Extended Data Fig. 6-1 and ANOVA results in Table 5). All fit parameters increased with neuropeptides compared with the decentralized control condition, indicating that LP was overall firing more spikes in longer bursts, consistent with the increased excitability seen in the *f–I* curves (Fig. 5). Neuropeptides increased the likelihood of LP rebound from inhibition and overall decreased the variability of the rebound parameters compared with the two unmodulated conditions (decentralized and wash). However, we observed a decrease of variability with increasing numbers of neuropeptides only for the fit parameters *t*_1/2_ and *k*, but not for the latency or the total number of spikes within a rebound burst (Fig. 6*D*, top row). The decrease of variability was present even when the parameters were not statistically different between the modulatory conditions.

10.1523/ENEURO.0167-24.2024.f6-1 Figure 6-1 All data for the analysis shown in Figure 6D. The Excel file contains four sheets, one for latency (in s) and one for each fit parameter (#spks, t_1/2_ (in s), slope factor (k)). In each sheet the first column is the unique identifier for each experiment. Column headers indicate the experimental conditions in the order of application: intact, decentralized (neuromodulatory inputs removed), mid (3 × 10^−8^ M) concentrations of PROC (P [mid]), PROC + CCAP (PC [mid]), PROC + CCAP + RPCH (PCR [mid]), wash. NaN indicates that the respective neuron or preparation was not active in that condition. Empty cells indicate that that neuromodulator combination was not applied to those experiments. Download Figure 6-1, XLS file.

**Table 5.** ANOVA results for 10 s rebound parameters

*N*	Parameter	Normality	Variance	Test statistic	df	res.	*p*
19	latency	Fail		*H* = 3.385	4		0.495
19	#spks	Pass	Pass	*F* = 8.665	4	77	**<0.001**
19	*t*_1/2_	Fail		*H* = 28.343	4		**<0**.**001**
19	slope factor (k)	Fail		*H* = 13.148	4		0.011

If tests for normality or equal variance failed, results are for ANOVA on ranks. Groups are decentralized, P [mid], PC [mid], PCR [mid], wash. *p* values smaller than *α* are printed in bold. *N*, number of animals; df, degrees of freedom; res, residual.

**Figure 7.**
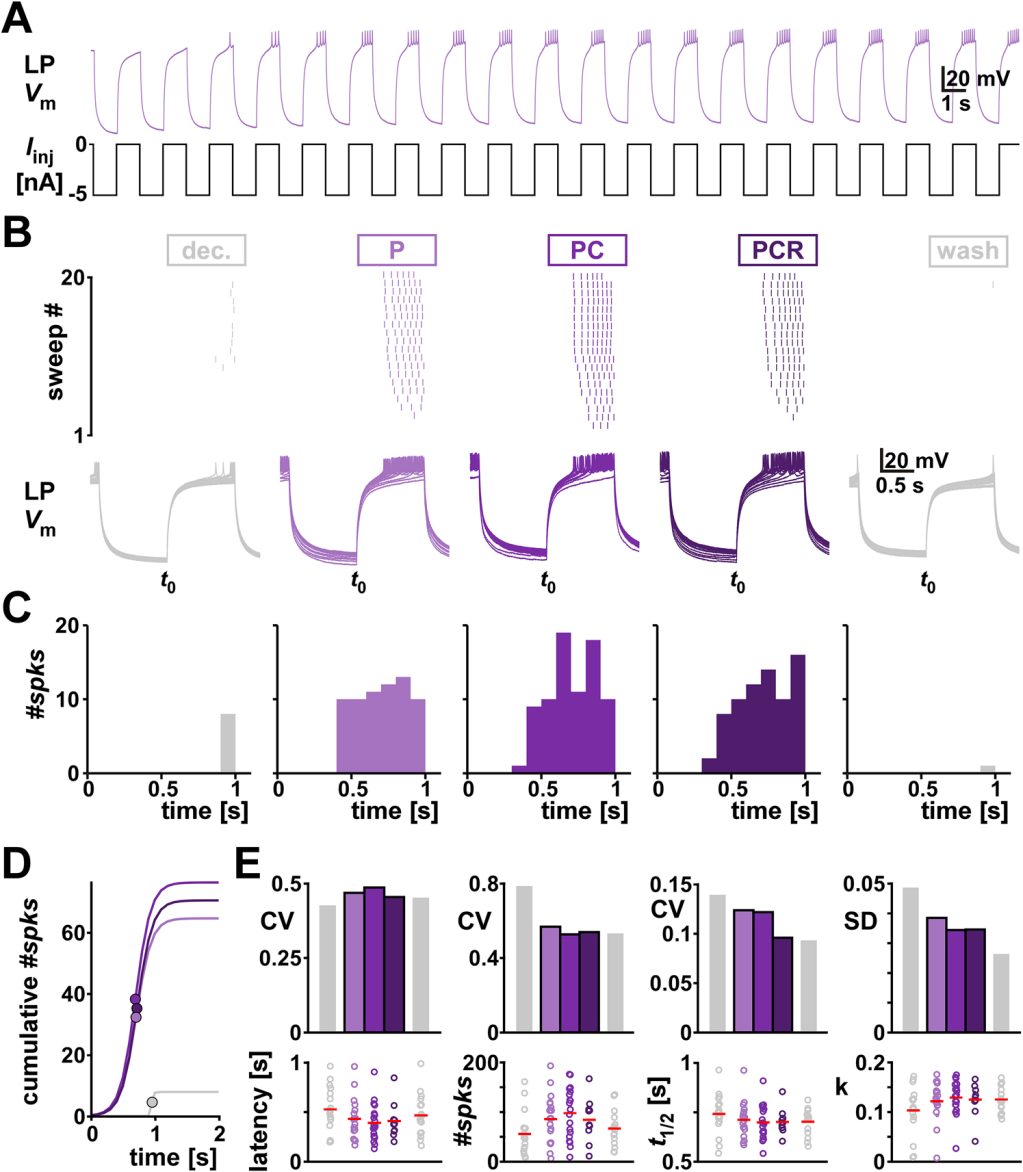
Comodulation does not reduce the interindividual variability of rebound from periodic inhibition on the single cell level. ***A***, One example of LP membrane potential in response to periodic hyperpolarization. ***B***, One example periodic rebound experiment in different modulatory conditions (color coded). The top panels show the spike raster plots for all 20 sweeps. The bottom panels show the intracellular voltage of all sweeps. *t*_0_ indicates the time point when LP was released from current injection. ***C***, Spike histogram across the last 10 sweeps in different modulatory conditions. ***D***, Sigmoid fits to the cumulative spike histograms. Dots indicate the sigmoid midpoint. ***E***, Latency and fit parameters (#*spks* = a, slope factor = *k* in Eq. 2) with the corresponding metric of variability (CV, or SD for interval data) for different modulatory conditions (color coded). Dots represent values from individual experiments; horizontal bars indicate the means. *N* = 19, except PCR *N* = 11. Statistical results in Table 6 indicate no significant difference between the means of the groups. Total modulator concentration for [mid]: 1–3 × 10^−8^ M for P and PC, 3 × 10^−8^ M for PCR.

In the intact pyloric circuit, LP is periodically inhibited by a group of pacemaker neurons and typical values of pyloric *F*_cycle_ range from 0.5 to 2 Hz. To mimic this effect, we modified the rebound protocol and hyperpolarized for 1 s every 2 s, repeated 20 times (Fig. 7*A*). As shown previously (Goaillard et al., 2010; Anwar et al., 2022; Schneider et al., 2022), LP rebound only reaches steady state after several cycles (Fig. 7*B*). Therefore, we only included the last 10 sweeps in our analysis and examined the same rebound parameters as before (Fig. 7*C*). In contrast to the 10 s rebound protocol, the application of neuromodulators at mid concentration did not significantly change any of the parameters with this faster protocol (Fig. 7*D*; detailed data are provided in Extended Data Fig. 7-1 and ANOVA results in Table 6). Furthermore, while variability did not increase with the addition of comodulators, any reduction seen was minor and did not recover after washing (Fig. 7*E*).

10.1523/ENEURO.0167-24.2024.f7-1 Figure 7-1 All data for the analysis shown in Figure 7E. The Excel file contains four sheets, one for latency (in s) and one for each fit parameter (#spks, t_1/2_ (in s), slope factor (k)).. In each sheet the first column is the unique identifier for each experiment. Column headers indicate the experimental conditions in the order of application: intact, decentralized (neuromodulatory inputs removed), mid (3 × 10^−8^ M) concentrations of PROC (P [mid]), PROC + CCAP (PC [mid]), PROC + CCAP + RPCH (PCR [mid]), wash. Latency is the latency (in s) over all five sweeps. NaN indicates that the respective neuron or preparation was not active in that condition. Empty cells indicate that that neuromodulator combination was not applied to those experiments. Download Figure 7-1, XLS file.

**Table 6.** ANOVA results for 1 s rebound parameters

*N*	Parameter	Normality	Variance	Test statistic	df	res.	*p*
19	latency	Fail		*H* = 5.329	4		0.249
19	#spks	Pass	Pass	*F* = 2.371	4	76	0.060
19	*t*_1/2_	Pass	Pass	*F* = 0.765	4	76	0.551
19	slope factor (k)	Fail		*H* = 4.068	4		0.397

If tests for normality or equal variance failed, results are for ANOVA on ranks. Groups are decentralized, P [mid], PC [mid], PCR [mid], wash. *p* values smaller than *α* are printed in bold. *N*, number of animals; df, degrees of freedom; res, residual.

### Peptide comodulation at saturating concentrations did not reduce variability of circuit activity attributes beyond the effects of a single peptide

So far, we have shown in this study that increasing the number of comodulatory neuropeptides at mid concentrations, but not at low concentrations, decreased the variability of several pyloric circuit rhythm attributes across animals. In contrast, at the single-neuron level, even though application of an individual peptide reduces variability of spike frequency and postinhibitory rebound properties, additional reduction of variability with comodulation is absent or ambiguous. This latter finding is consistent with previous findings that, at saturating concentrations (≥1 µM), the addition of a second comodulatory peptide does not further reduce neuronal output variability (Schneider et al., 2022). However, at saturating concentrations, convergent modulators may also occlude one another's actions on a single neuron but may have distinct actions on neurons that do not express receptors for all these modulators. This raised the question of whether comodulation at saturating levels reduces variability of the full circuit output attributes.

**Figure 8.**
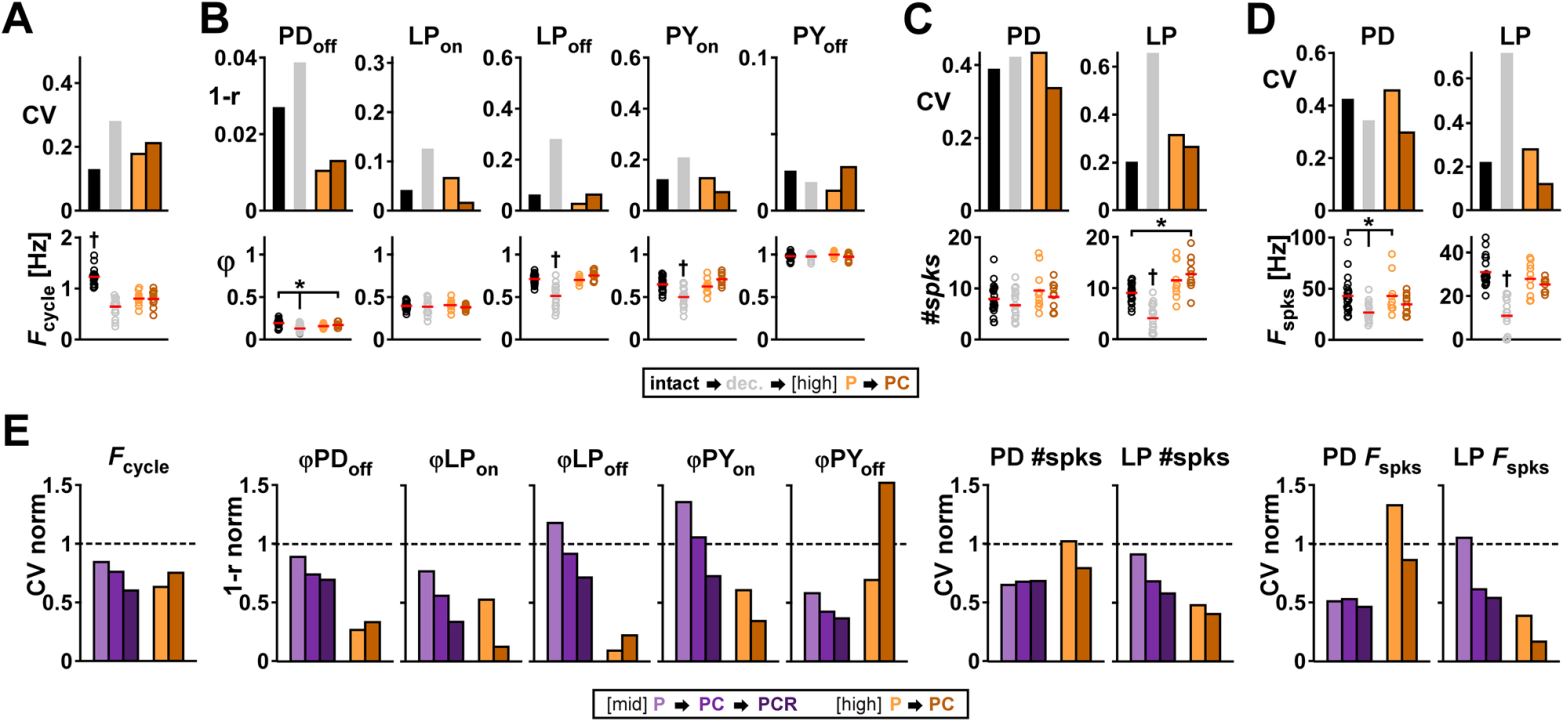
Neuropeptides at high concentration reduce the interindividual variability of pyloric rhythm parameters on the circuit output level. ***A***, *F*_cycle_ and the corresponding CV under different modulatory conditions (intact, dec., P and PC; color coded) at [high]: 10^−6^ M concentration. ***B***, Burst start (on) and termination (off) and the corresponding circular variance (1 − *r*; see Materials and Methods) under different [high] modulatory conditions (same color coding as panel ***A***). ***C***, Number of spikes per burst (#spks) and corresponding CV for each type of neuron at different [high] modulatory conditions (same color coding as panel ***A***). ***D***, Average spike frequency (*F*_spks_) within a burst and corresponding CV for each type of neuron at each [high] modulatory condition (same color coding as panel ***A***). ***E***, The CV or circular variance (1−*r*) from panels ***A***–***D*** for conditions P and PC are shown normalized to the corresponding CV of 1 − *r* for the decentralized (dec., dashed line) condition. In each panel, the purple bars show the corresponding variability (also normalized to the corresponding dec. value) at [mid] P, PC, and PCR (from Figs. 2–4), for comparison. Separate datasets for P and PC, *N* = 10 each. Individual dots represent data from individual experiments; red bars indicate the mean value. Asterisks indicate pairwise significant differences between two groups or the group indicated with the longer line and those indicated with shorter lines (Dunn's post hoc test; *p* ≤ 0.05). The groups indicated with daggers are significantly different from all other groups in that panel. All other pairwise comparisons were not statistically significant. Statistical results in Table 7. Total modulator concentration for [high]: 10^−6^ M.

We addressed this question with the same two modulators (PROC and CCAP) used in the Schneider et al. (2022) study. Figure 8 summarizes the results of applying P and PC at a total concentration of 1 µM to the decentralized preparation (detailed data are provided in Extended Data Fig. 8-1 and ANOVA results in Table 7). In contrast to the experiments at low and mid concentrations, we only used experiments where the pyloric rhythm continued when the preparation was decentralized. In these experiments, *F*_cycle_ was significantly higher in the intact condition (Fig. 8*A*), and decentralization impacted the off phases of PD and LP and the on phase of PY (Fig. 8*B*). For frequency and phases, variability across individuals was lower with modulators than without (Fig. 8*A*,*B*). Variability metrics for spike number (Fig. 8*C*) and spike frequency (Fig. 8*D*) within a burst decreased with modulators for the LP neuron but not for the PD neuron. Overall, at these saturating concentrations, the variability across animals was in the same range as in the intact condition.

10.1523/ENEURO.0167-24.2024.f8-1 Figure 8-1 All data for the analysis shown in Figure 9. The Excel file contains ten sheets, one for *F*_cycle_ (in Hz) and one for each neuron type and rhythm parameter (PD_off_, LP_on_, LP_off_, PY_on_, PY_off_, PD_#spks_, LP_#spks_, PY_#spks_, PD_F_spks_ LP_F_spks_, PY_F_spks_). In each sheet the first column is the unique identifier for each experiment. crab_1, crab_2 denotes the two preparations in the same dish from which we recorded simultaneously. Data columns show the average cycle frequency in Hz, phase on and off, number of spikes (#_spks_) and spike frequency (*F*_spks_) in Hz over 30  s recordings (at least 10 bursts) for each experiment. Column headers indicate the experimental conditions in the order of application: intact, decentralized (neuromodulatory inputs removed), mid (3 × 10^−8^ M) concentrations of PROC (P [mid]), PROC + CCAP (PC [mid]), PROC + CCAP + RPCH (PCR [mid]), wash. NaN indicates that the respective neuron or preparation was not active in that condition. Empty cells indicate that that neuromodulator combination was not applied to those experiments. Download Figure 8-1, XLS file.

**Table 7.** ANOVA results for pyloric rhythm attributes at high modulator concentrations

*N*	Attribute	Normality	Variance	Test statistic	df	res.	*p*
20	*F*_cycle_	Pass	Pass	*F* = 43.965	3	54	**<0**.**001**
20	PD_off_	Pass	Pass	*F* = 9.373	3	54	**<0**.**001**
20	LP_on_	Pass	Fail	*H* = 1.457	3		0.692
20	LP_off_	Pass	Fail	*H* = 30.555	3		**<0**.**001**
20	PY_on_	Pass	Pass	*F* = 14.001	3	54	**<0**.**001**
20	PY_off_	Fail		*H* = 2.913	3		0.405
20	PD #spks	Fail		*H* = 4.285	3		0.232
20	PD *F*_spks_	Fail		*H* = 13.449	3		**0**.**004**
20	LP #spks	Pass	Pass	*F* = 28.269	3	54	**<0**.**001**
20	LP *F*_spks_	Pass	Pass	*F* = 30.227	3	54	**<0**.**001**

If tests for normality or equal variance failed, results are for ANOVA on ranks. Groups are intact, decentralized, P [high], PC [high], wash. *p* values smaller than *α* are printed in bold. *N*, number of animals; df, degrees of freedom; res, residual.

To compare the effect of [high] versus [mid] concentrations, we normalized the variability in the presence of each modulator to the variability of the decentralized (control) condition for that attribute and plotted these values side by side (Fig. 8*E*). For phases and LP spike attributes, the variability at high concentrations was lower than that at mid concentrations, but this concentration-dependent effect was not present for cycle frequency or PD spike attributes. Notably, the addition of a comodulator (PC vs P) did not further reduce variability in a consistent manner at high concentrations as it did at mid concentrations. Taken together, these results show that although increasing the modulator concentration may decrease variability of some attributes, comodulation at saturating concentrations does not consistently reduce variability at the network level in the manner that mid-concentration comodulation does.

## Discussion

### How could convergent comodulation reduce interindividual variability?

Generally, there are two possibilities for how convergent peptide comodulation of the pyloric circuit could lead to the reduction of circuit output variability. First, variability may be reduced at the single-neuron level, for instance, because comodulation produces more consistent levels of *I*_MI_, as some neuron types only have receptors for a subset of these peptides (Swensen and Marder, 2001; Garcia et al., 2015). Alternatively, reduction in interindividual variability could be a circuit-level phenomenon.

A recent study examined the interindividual variability of single-neuron responses to excitatory neuromodulation applied at saturating levels (Schneider et al., 2022). That study showed that variability was greatly reduced in the presence of PROC, yet the reduction of variability was the same in the presence of PROC alone or with the combined application of PROC and CCAP, indicating that comodulation did not reduce variability compared with a single modulator. In the current study, we found that mid-level concentration peptide modulation also reduced variability of the isolated LP neuron outputs and, as in the Schneider et al. (2022) study, there was no additional reduction of variability by comodulation (Figs. 5–7). Convergent modulator effects depend on both concentration and receptor expression. If two modulators targeting the same downstream pathway are applied at saturating concentrations, occlusion occurs and comodulation will have little additional effect compared with one modulator. However, at subsaturation levels, additive application will increase excitability, which is why we used constant total concentrations. If the activation pathway toward the common target ion channel is through different receptors, interindividual variability may be decreased by comodulation because variability in the different receptor populations averages out.

Yet, increasing the number of modulators while keeping the total concentration constant is an imperfect way to keep the overall activation of a circuit similar for several reasons. First, receptor activation depends on a dose–response curve that is typically sigmoidal on a log scale. Therefore, even for two receptors with identical expression levels and concentration dependence, application of a single modulator at any concentration results in a lower level of overall G-protein activation than coapplication of two modulators at half that concentration. Second, receptor expression and dose–response curves vary across neuron types (Garcia et al., 2015), rendering consequences of coapplications further distinct from simple linearly additive effects. An extreme version of this is when a single neuron only expresses the receptor to one of two coapplied modulators, resulting in less activation during coapplication than during single application. Third, coapplication of neuropeptides may lead to sublinear combined *I*_MI_ activation because of potential inhibitory interactions in the signaling pathways (Li et al., 2018; Dickinson and Powell, 2023). For these reasons, we do not claim that keeping the total concentrations constant ensures equal overall circuit activation. However, it is a practical way to keep the circuit in an activation range that is not subject to saturation effects, which would more easily occur with additive applications.

Still, comodulation can lead to increasingly consistent modulation across multiple components at the circuit level, both with additive and constant total concentrations. Both increase in excitability and consistency in activation can decrease interindividual variability. Thus, our findings indicate that the comodulation-mediated reduction of variability is unlikely to emerge at the level of individual neurons and therefore it likely emerges at the circuit level. There are many ways such circuit-level actions of modulators can arise. For instance, modulation of a single neuron may have effects that are restricted to that neuron or, conversely, reverberate through other circuit neurons, as was demonstrated in a computational study exploring the effects of hub neurons in a network (Gutierrez and Marder, 2014). Additionally, the excitatory neuropeptides also influence synaptic efficacy and dynamics in this circuit (Thirumalai et al., 2006; Atamturktur and Nadim, 2011; Zhao et al., 2011; Li et al., 2018), which in turn influences the activities of all circuit neurons. Finally, interactions between nonlinear properties of bursting neurons connected with recurrent synapses that have short-term dynamics could give rise to effects that are not present in isolated neurons or feedforward circuits (Tsodyks et al., 2000; Yuste, 2015; Akcay et al., 2018).

### Modulator action to shape specific circuit outputs versus reduction of variability

Neuromodulators play a crucial role in the flexibility of neural circuits through acting on a wide range of (mainly metabotropic) receptors to alter levels of ionic currents and even ion transporters, thereby greatly influencing neuronal excitability (Marder, 2012). The same receptors also act on synaptic release mechanisms and neurotransmitter receptors to change synaptic efficacy, alter the sensitivity of neurons to input, and modify synaptic plasticity (Nadim and Bucher, 2014). This dynamic modulation allows neural circuits to adapt and reorganize their connectivity patterns, facilitating flexible and adaptive responses based on context, such as response to stimuli, circadian rhythmicity, or intrinsic states such as arousal and stress (Likhtik and Johansen, 2019; McCormick et al., 2020; Zolin et al., 2021). Extensive research on neuromodulator actions has shown that their effects on circuit components can shape circuit output in a relatively consistent manner to meet these contextual demands (Nadim and Bucher, 2014).

Here, we are proposing a somewhat different role for neuromodulation, in that convergent comodulation could have significant impact on ensuring the consistent output of neural circuits, particularly when faced with substantial interindividual variability. Our findings do not counter, but rather complement, the classical role for neuromodulators in providing flexibility of circuit output. For instance, even with multiple modulatory inputs, the presence of a single modulator at high concentration could shift circuit output (i.e., the mean values of the output attributes) toward a modulator-specific pattern. In contrast, what we propose to result from mid-concentration comodulation is not a new target mean value for the output attributes but simply reducing the variability of the output without necessarily changing these mean values. This is an effect of comodulation, not an effect of modulation by a single chemical. Moreover, as our results show, this is not a concentration-dependent effect, since in our experiments we ensured that the total concentration remained constant when 1, 2, or 3 modulators were applied.

Modulatory actions clearly depend on the modulator concentrations and, in the case of modulatory neurotransmitters, on the cohort of cotransmitters and the specific release targets (Nusbaum et al., 2001, 2017) which were not considered in the current study. At very low concentrations, threshold effects may produce, rather than reduce, interindividual variability. The low concentration of 1 nM is close to threshold of *I*_MI_ activation for PROC and CCAP (Li et al., 2018). Since we kept the total modulator concentration constant, inconsistent changes in variability at low concentrations could be due to these threshold effects. At the other extreme, saturating concentrations may also have somewhat different effects on variability. Overall, the interindividual variability of the pyloric circuit output attributes were consistently lower at high peptide concentrations compared with mid concentrations (Fig. 8*E*). However, comodulation at high concentrations did not consistently reduce variability compared with a single modulator. This is likely in part due to the saturation of neuropeptide receptors since receptor variability is also high across animals (Schneider et al., 2022). It therefore appears that the comodulatory actions that result in the reduction of interindividual variability occur at some limited range of concentrations, perhaps consistent with tonic levels of these modulators in biological conditions.

### Conclusions

The presence of interindividual variability of ion channel expression and synaptic properties poses the important question of how such variability may be constrained so that neural circuits could produce biologically meaningful output. The hypothesis that neuromodulators may be involved in compensating for interindividual variability well precedes the current study (Hamood and Marder, 2014). We have modified this hypothesis to propose that it is the combined overlapping tonic presence of modulatory action that could provide a reasonable answer to constraining interindividual variability at the neural systems level. Considering that all neural systems are constantly targeted by actions of multiple modulators whose receptors have considerable overlapping subcellular actions (Doi and Ramirez, 2008; Harris-Warrick, 2011; Marder, 2012; Russo, 2017; McCormick et al., 2020), our findings justify the exploration of similar mechanisms in other neural circuits across animals.

## Synthesis

Reviewing Editor: Arvind Kumar, KTH Royal Institute of Technology Decisions are customarily a result of the Reviewing Editor and the peer reviewers coming together and discussing their recommendations until a consensus is reached. When revisions are invited, a fact-based synthesis statement explaining their decision and outlining what is needed to prepare a revision will be listed below. The following reviewer(s) agreed to reveal their identity: Ronald Calabrese.

Synthesis

The two reviewers agree that the manuscript addresses an important question about how mixtures of neuromodulators may affect the network dynamics and function.

The main claim of the paper is that a mixture of the neuromodulators is more effective in reducing the variability of the pyloric rhythm generator. However, reviewers are not completely convinced by this claim. This is partly because of the way the data has been presented. The reviewers also pointed out that the computational model does not really add anything to the manuscript. The results do not match up and we do not get any mechanistic understanding of neuromodulation. This is largely due to the choice of the model and focus only on a single parameter when it is obvious that neuromodulation affects both synaptic and neuronal properties.

After consultation reviewers think that two main modifications are needed in the manuscript:

1. Remove the computational model

2. Re-render the data shown in the figure 8 as suggested by the reviewers.

Besides, the reviewers have raised several other comments.I have appended the detailed comments which should also be addressed in the revised version of the manuscript.

Reviewer #1

This paper describes a well conducted study using standard electrophysiological techniques that determines the effects of peptide co-modulation on interindividual output variability in a well character CPG network, the pyloric circuit of the crab stomatogastric nervous system. This experimental work is supplemented by ensemble modeling of the pyloric network to test a simple hypothesis concerning the experimental results obtained. The data is extensive (vast even) and thoughtfully analyzed with appropriate metrics and statistical analyses. The experiments clearly show that at an intermediate total concentration of co-modulators reduces interindividual variability of output attributes compared to a similar concentration of a single modulator. The Figures are clear and give the essential data. Extended data is supportive, and I think adds to the paper, but is mainly presented as spreadsheets and would be more useful if also presented as figures or well-formatted tables. I have some suggested revisions.

Abstract:

Significance Statement:

1. "We use two general facts about neuromodulation to show that convergent comodulation at tonic mid-concentration levels reduces interindividual variability of neural circuit output. These facts are that 1) Multiple neuromodulators can have convergent actions on the same subcellular substrates and 2) Neurons express different neuromodulator receptors at different levels." This seems very awkward, backward even.

Figures:

2. Only 2 of the extended data figures cited in the text were actually presented as figures, the rest are just spreadsheets of numbers.

3. Figure 4C is not mentioned in the text, and I saw no reason for it. Delete?

4. I suggest that you make Figure 9G a separate figure so that it can be easily seen and interpreted; it seems key to the interpretation of the modeling.

Introduction:

1. Consider citing -- doi: 10.1523/JNEUROSCI.5072-10.2011; doi: 10.1152/jn.00856.2013; doi:10.7554/eLife.31123.

2. Line 41: missing in before dysfunctional.

3. Lines 73-74: I cannot follow the end of this sentence. Please rewrite.

Methods (very difficult to follow in some places):

1. Current Injection: In Methods and in the Results no fewer than 4 words are used to describe the same set of current injections: sweeps, injections, pulses, steps. Using one word would make the paper easier to read because the reader would not be wondering if the change in wording reflects some fundamental difference.

2. Lines 114-115: "In total, we obtained four different datasets, each with intact and decentralized conditions in addition to:" I can't follow this, please rewrite.

3. In Methods you introduce φ to represent phase and use it in the Figures but later in Results (line 460) you introduce ϕ to represent phase. Please use consistent symbols.

4. Line 205: What is the difference between a neurite and a dendrite in a unipolar neuron like PD?

Results:

Major

1. In introduction you clearly state "PROC activates IMI in AB, PD, LP, and most PY neurons, while both CCAP and RPCH activate IMI only in AB and LP neurons (Fig. 2A) (Swensen and Marder 2001)." It then seems clear to me that your modeling exercise is doomed from the start to not reflect biological reality because you artificially separate the PROC grouping, posit a modulator that solely affects PY which does not exist, and fail to separate PD and AB as modulator target cells (e.g., RPCH). I did not take away much from the modeling and Figure 9 is difficult to follow. Can you better rationalize your choice of modulator targets to justify this modeling exercise? It seems to me you are just hypothesizing that more target cells is how co-modulation works whereas in the crab STG the baseline modulator PROC hits everyone and the other peptides fine tune this blanket approach.

Minor

1. Line 255: "soup". Maybe mixture?

2. Lines 391-392: I can't follow this sentence, please rewrite.

Discussion:

1. Line 607: "goldilocks". Restricted?

Reviewer #2

In the present article, the authors attempt to demonstrate that co-modulation of different types of neurons in a small network reduces the variability of network output. The study is performed on the stomatogastric nervous system of the crab, and more specifically on the pyloric network. The authors then use different mixes of neuropeptides known to modulate different neurons in the network (Proctolin, CCAP and RPCH), mix them at low, mid and high concentrations and measure changes induced in the animal-to-animal (interindividual) variability in circuit output parameters. The authors also use database modeling of a simplified pyloric network to evaluate the generic value of their experimental results.

In general, the article is very well written, the experiments, modeling and analyses are very well performed, and the statistics appropriately chosen and used. Moreover, the idea that co-modulation may reduce variability is very appealing, and thus the question asked is relevant and important. However, the results are not as convincing as the title may promise it. In particular, the experiment illustrated in Figure 8, although not presented as such, seems to demonstrate that high concentration of a single peptide (here Proctolin) is as efficient as the co-application of 3 peptides at mid-concentrations to reduce variability of circuit output, therefore contradicting the main conclusion of the article. In addition, the computational model is not made to simulate the exact effect of the peptides used in the experiments (although addressing the effect of differentially modulating several neuronal targets), and thus provides results that do not allow the authors to draw a definitive conclusion about the effects of co-modulation on output variability. In conclusion, although the question addressed in this study is really important and well treated, I am not totally convinced that the results support the conclusions drawn by the authors.

MAIN CONCERNS

1) The main point of the article is to demonstrate that co-modulation (at mid-concentrations) is more efficient than the application of single modulators at high concentration to reduce inter-individual variability. In particular, testing high concentration of P and PC and comparing the effect to mid-concentrations is supposed to support this claim. Unfortunately, the results presented in Figure 8 argue against this conclusion. First, I would suggest two modifications to Figure 8 in order to have a fair comparison between the effects of high and mid-concentrations: i) as there are large differences in the control values of most parameters (see the black bars in Figure 8 compared to Figures 2, 3, 4) the relative magnitudes of the effects of high concentrations of P and PC (shades of ocre-filled bars) and mid-concentrations of the same modulators (empty purple bars) cannot be compared. I would suggest putting the bar plots from mid-concentrations and high concentrations side by side, with the control values at the same level, allowing to really compare the magnitudes of effects of the modulators ii) Second, to support the idea that co-modulation reduces variability, it would be very useful to present the results of PCR at mid-concentration together with the results of P and PC at high concentrations.

Notwithstanding the presentation of Figure 8, the results with high concentration of P and PC argue against the enhanced reduction of variability with more modulators (PCR at mid-concentration). Indeed, most of the parameters seem to show a stronger reduction of variability when exposed to high concentrations of P or PC (Figure 8). Maybe this result is due to the fact that the authors chose Proctolin as the modulator to apply at high concentration, and that Proctolin targets all the neuronal types in the pyloric network (whereas CCAP and RPCH only act on AB and LP). Maybe the experiment could be done with CCAP (or RPCH) alone in order to prove this point. Proctolin could be used as a second or third modulator instead of first in that case. Alternatively or additionally, I was surprised by the choice of CCAP and RPCH as second and third modulators as these two modulators target the same population of neurons (except for specific synapses, but data from the literature are missing to support this point). Why not use a modulator such as TNRNFLRF, which acts on AB, LP and PYs but not on PD ? In fact, the results presented in Figure 8 and their comparison with the PCR at mid-concentrations could be interpreted mainly as a dose effect because higher concentration of a single peptide seems to decrease variability as efficiently as mid-concentrations of several peptides (but once again, the choice of proctolin, targeting all the neuronal types may be responsible for this confusion).

2) My second main concern is about the outcome of the modeling part of the study. In my opinion, it is difficult to draw definitive conclusions from the modeling performed by the authors. First, the authors decided to build a model that does not exactly reproduce the effect of the modulators applied in the experiments. Instead of simulating an increase in IMI in the specific biological targets of P, C and R (including the synaptic targets too), the authors simulate an increase in isolated targets (AB/PD, LP or PY). In this model, the authors find that increasing gMI tends to decrease variability in circuit output, although a maximal decrease for mid-concentrations (mid-range of gMI) is not systematically observed. Hence the authors conclude that this simplified version does not reproduce the overall results of the experiments, which suggest more convincingly that co-modulation reduces variability. I agree with the authors, but it is difficult to know whether co-modulation in general is much better at reducing variability (see point 1) or whether the oversimplification of the model of neuromodulation is responsible for the lack of positive results with the model. In the end, one cannot say whether the model really tells us anything about the generic principles and the benefits of co-modulation.

Minor points

1) The author focus on interindividual variability, but never mention the cycle-to-cycle variability within the same animal under the unmodulated or co-modulated conditions. Obviously this is a different question, but it could be interesting to report this variability and see whether co-modulation tends to increase the reliability of the pyloric rhythm (decreasing cycle-to-cycle variability in circuit output parameters).

2) I want to thank the authors for providing all the raw data and precise statistics in supplementary files. However, all statistical results have to be searched for within these many supplementary files. For the readibility of the manuscript (even though there are many conditions and thus many pairwise comparisons), it would be really nice to have some graphical reports of these statistics directly in the main figures (brackets and asterisks for instance on the scatter plots).

3) I had much trouble understanding how the sigmoidal fits of rebound spikes (Figures 6 and 7) were obtained, since the experimental points constraining the fit do not appear on the figure. Maybe the authors should represent the experimental points so that we can have a better idea of the match between the fit and the experiment.

4) Page 2, line 41: "... and significant variability results IN dysfunctional output".

5) Page 12, line 353: the terme slope factor is used in the text while the term scale factor is also to describe the same factor alpha. For clarity, the authors should chose one term and keep it throughout the manuscript.

## Author Response

We are extremely grateful to the two reviewers and the Reviewing Editor for their helpful comments. We have made every effort to address all comments. Additionally, we have removed the modeling component of the study since it clearly led to more confusion than clarification. We address the points of the reviewers below in detail.

Sincerely, The Authors Synthesis of Reviews:

Computational Neuroscience Model Code Accessibility Comments for Author (Required):

Model is available on github Synthesis Statement for Author (Required):

Synthesis The two reviewers agree that the manuscript addresses an important question about how mixtures of neuromodulators may affect the network dynamics and function.

The main claim of the paper is that a mixture of the neuromodulators is more effective in reducing the variability of the pyloric rhythm generator. However, reviewers are not completely convinced by this claim. This is partly because of the way the data has been presented. The reviewers also pointed out that the computational model does not really add anything to the manuscript. The results do not match up and we do not get any mechanistic understanding of neuromodulation. This is largely due to the choice of the model and focus only on a single parameter when it is obvious that neuromodulation affects both synaptic and neuronal properties.

After consultation reviewers think that two main modifications are needed in the manuscript:

1. Remove the computational model 2. Re-render the data shown in the figure 8 as suggested by the reviewers.

Besides, the reviewers have raised several other comments.I have appended the detailed comments which should also be addressed in the revised version of the manuscript.

Reviewer #1 This paper describes a well conducted study using standard electrophysiological techniques that determines the effects of peptide co-modulation on interindividual output variability in a well character CPG network, the pyloric circuit of the crab stomatogastric nervous system. This experimental work is supplemented by ensemble modeling of the pyloric network to test a simple hypothesis concerning the experimental results obtained. The data is extensive (vast even) and thoughtfully analyzed with appropriate metrics and statistical analyses. The experiments clearly show that at an intermediate total concentration of co-modulators reduces interindividual variability of output attributes compared to a similar concentration of a single modulator. The Figures are clear and give the essential data. Extended data is supportive, and I think adds to the paper, but is mainly presented as spreadsheets and would be more useful if also presented as figures or well-formatted tables. I have some suggested revisions.

Abstract:

Significance Statement:

1. "We use two general facts about neuromodulation to show that convergent comodulation at tonic mid-concentration levels reduces interindividual variability of neural circuit output. These facts are that 1) Multiple neuromodulators can have convergent actions on the same subcellular substrates and 2) Neurons express different neuromodulator receptors at different levels." This seems very awkward, backward even.

We rewrote the significance statement.

Figures:

2. Only 2 of the extended data figures cited in the text were actually presented as figures, the rest are just spreadsheets of numbers.

Our understanding is that the extended data could include data tables supporting what is shown in a figure, yet these tables related to Figure X should be labeled Figure X-1, X-2, etc. Nevertheless we have now moved the statistical data into tables in the main text.

3. Figure 4C is not mentioned in the text, and I saw no reason for it. Delete? This was an oversight on our part. Considering the figure summarizes the results of Figure 4, we decided to keep it and explain it properly.

4. I suggest that you make Figure 9G a separate figure so that it can be easily seen and interpreted; it seems key to the interpretation of the modeling.

Figure 9 and all modeling results have been removed as requested by the consultation remark above.

Introduction:

1. Consider citing -- doi: 10.1523/JNEUROSCI.5072-10.2011; doi: 10.1152/jn.00856.2013; doi:10.7554/eLife.31123.

Done.

2. Line 41: missing in before dysfunctional.

Corrected.

3. Lines 73-74: I cannot follow the end of this sentence. Please rewrite.

Fixed.

Methods (very difficult to follow in some places):

1. Current Injection: In Methods and in the Results no fewer than 4 words are used to describe the same set of current injections: sweeps, injections, pulses, steps. Using one word would make the paper easier to read because the reader would not be wondering if the change in wording reflects some fundamental difference.

Fixed.

2. Lines 114-115: "In total, we obtained four different datasets, each with intact and decentralized conditions in addition to:" I can't follow this, please rewrite.

Fixed.

3. In Methods you introduce φ to represent phase and use it in the Figures but later in Results (line 460) you introduce ϕ to represent phase. Please use consistent symbols.

Fixed.

4. Line 205: What is the difference between a neurite and a dendrite in a unipolar neuron like PD? Fixed.

Results:

Major 1. In introduction you clearly state "PROC activates IMI in AB, PD, LP, and most PY neurons, while both CCAP and RPCH activate IMI only in AB and LP neurons (Fig. 2A) (Swensen and Marder 2001)." It then seems clear to me that your modeling exercise is doomed from the start to not reflect biological reality because you artificially separate the PROC grouping, posit a modulator that solely affects PY which does not exist, and fail to separate PD and AB as modulator target cells (e.g., RPCH). I did not take away much from the modeling and Figure 9 is difficult to follow. Can you better rationalize your choice of modulator targets to justify this modeling exercise? It seems to me you are just hypothesizing that more target cells is how co-modulation works whereas in the crab STG the baseline modulator PROC hits everyone and the other peptides fine tune this blanket approach.

Figure 9 and all modeling results have been removed as requested by the consultation remark above.

Minor 1. Line 255: "soup". Maybe mixture? Changed.

2. Lines 391-392: I can't follow this sentence, please rewrite.

Fixed.

Discussion:

1. Line 607: "goldilocks". Restricted? Changed.

Reviewer #2 In the present article, the authors attempt to demonstrate that co-modulation of different types of neurons in a small network reduces the variability of network output. The study is performed on the stomatogastric nervous system of the crab, and more specifically on the pyloric network. The authors then use different mixes of neuropeptides known to modulate different neurons in the network (Proctolin, CCAP and RPCH), mix them at low, mid and high concentrations and measure changes induced in the animal-to-animal (interindividual) variability in circuit output parameters. The authors also use database modeling of a simplified pyloric network to evaluate the generic value of their experimental results.

In general, the article is very well written, the experiments, modeling and analyses are very well performed, and the statistics appropriately chosen and used. Moreover, the idea that co-modulation may reduce variability is very appealing, and thus the question asked is relevant and important. However, the results are not as convincing as the title may promise it. In particular, the experiment illustrated in Figure 8, although not presented as such, seems to demonstrate that high concentration of a single peptide (here Proctolin) is as efficient as the co-application of 3 peptides at mid-concentrations to reduce variability of circuit output, therefore contradicting the main conclusion of the article. In addition, the computational model is not made to simulate the exact effect of the peptides used in the experiments (although addressing the effect of differentially modulating several neuronal targets), and thus provides results that do not allow the authors to draw a definitive conclusion about the effects of co-modulation on output variability. In conclusion, although the question addressed in this study is really important and well treated, I am not totally convinced that the results support the conclusions drawn by the authors.

MAIN CONCERNS 1) The main point of the article is to demonstrate that co-modulation (at mid-concentrations) is more efficient than the application of single modulators at high concentration to reduce inter-individual variability. In particular, testing high concentration of P and PC and comparing the effect to mid-concentrations is supposed to support this claim. Unfortunately, the results presented in Figure 8 argue against this conclusion. First, I would suggest two modifications to Figure 8 in order to have a fair comparison between the effects of high and mid-concentrations: i) as there are large differences in the control values of most parameters (see the black bars in Figure 8 compared to Figures 2, 3, 4) the relative magnitudes of the effects of high concentrations of P and PC (shades of ocre-filled bars) and mid-concentrations of the same modulators (empty purple bars) cannot be compared. I would suggest putting the bar plots from mid-concentrations and high concentrations side by side, with the control values at the same level, allowing to really compare the magnitudes of effects of the modulators ii) Second, to support the idea that co-modulation reduces variability, it would be very useful to present the results of PCR at mid-concentration together with the results of P and PC at high concentrations.

Notwithstanding the presentation of Figure 8, the results with high concentration of P and PC argue against the enhanced reduction of variability with more modulators (PCR at mid-concentration). Indeed, most of the parameters seem to show a stronger reduction of variability when exposed to high concentrations of P or PC (Figure 8). Maybe this result is due to the fact that the authors chose Proctolin as the modulator to apply at high concentration, and that Proctolin targets all the neuronal types in the pyloric network (whereas CCAP and RPCH only act on AB and LP). Maybe the experiment could be done with CCAP (or RPCH) alone in order to prove this point. Proctolin could be used as a second or third modulator instead of first in that case. Alternatively or additionally, I was surprised by the choice of CCAP and RPCH as second and third modulators as these two modulators target the same population of neurons (except for specific synapses, but data from the literature are missing to support this point). Why not use a modulator such as TNRNFLRF, which acts on AB, LP and PYs but not on PD ? In fact, the results presented in Figure 8 and their comparison with the PCR at mid-concentrations could be interpreted mainly as a dose effect because higher concentration of a single peptide seems to decrease variability as efficiently as mid-concentrations of several peptides (but once again, the choice of proctolin, targeting all the neuronal types may be responsible for this confusion).

With many apologies to the reviewer for the confusion we have caused, the point of this study is not that comodulation at mid-concentration reduces variability better than any single modulator at high concentration. Although we never say such a thing in the manuscript, it is easy to see why the reviewer got this impression. Please allow us to clarify. When applied at high concentration, any one of the excitatory peptides produces a pyloric rhythm with relatively low inter-animal variability. This is supported by numerous studies from many labs (including the Marder, Dickinson and Nusbaum labs). However, each peptide modulator at saturating concentration also carves out a specific stereotypical pattern of activity. This is the result that supports the primary role of modulators (which we cover in our Discussion section 6.3 - now 6.2). In other words, each modulator applied at high concentration shifts the mean value of a circuit output attribute towards a specific value (and, as the reviewer has observed, also reduces variability around this new target mean value). However, adding another modulator (comodulation) at high concentration does not necessarily reduce variability further, but mainly shifts the mean of the output attribute toward another target point (to produce a different stereotypical pattern).

In contrast, what we propose to result from mid-concentration comodulation is not a new target mean value for the output attributes, but simply reducing the variability of the output without necessarily changing these mean values. The output attributes do not necessarily change by adding more modulators at mid concentration, but the variability of the attribute is reduced as more (convergent) modulator types are added. This is an effect of comodulation, not an effect of modulation by a single peptide. Notably, this is not a concentration-dependent effect, since in our experiments we ensured that the total concentration remained constant when 1, 2 or 3 modulators were applied.

We hope this clarifies the confusion that we had caused. We have now added paragraphs to both the introduction and the discussion to clarify this important distinction for the readers.

We have also modified Figure 8 to allow for a fair comparison between mid- and high-concentrations as the reviewer suggests.

2) My second main concern is about the outcome of the modeling part of the study. In my opinion, it is difficult to draw definitive conclusions from the modeling performed by the authors. First, the authors decided to build a model that does not exactly reproduce the effect of the modulators applied in the experiments. Instead of simulating an increase in IMI in the specific biological targets of P, C and R (including the synaptic targets too), the authors simulate an increase in isolated targets (AB/PD, LP or PY). In this model, the authors find that increasing gMI tends to decrease variability in circuit output, although a maximal decrease for mid-concentrations (mid-range of gMI) is not systematically observed. Hence the authors conclude that this simplified version does not reproduce the overall results of the experiments, which suggest more convincingly that co-modulation reduces variability. I agree with the authors, but it is difficult to know whether co-modulation in general is much better at reducing variability (see point 1) or whether the oversimplification of the model of neuromodulation is responsible for the lack of positive results with the model. In the end, one cannot say whether the model really tells us anything about the generic principles and the benefits of co-modulation.

Figure 9 and all modeling results have been removed as requested by the consultation remark above.

Minor points 1) The author focus on interindividual variability, but never mention the cycle-to-cycle variability within the same animal under the unmodulated or co-modulated conditions. Obviously this is a different question, but it could be interesting to report this variability and see whether co-modulation tends to increase the reliability of the pyloric rhythm (decreasing cycle-to-cycle variability in circuit output parameters).

We added the data for cycle-to-cycle variability in the paper (Table 3) and discussed these results in a paragraph.

2) I want to thank the authors for providing all the raw data and precise statistics in supplementary files. However, all statistical results have to be searched for within these many supplementary files. For the readibility of the manuscript (even though there are many conditions and thus many pairwise comparisons), it would be really nice to have some graphical reports of these statistics directly in the main figures (brackets and asterisks for instance on the scatter plots).

We have added the statistical differences into the graphs and now report the statistics as tables not as extended figure data.

3) I had much trouble understanding how the sigmoidal fits of rebound spikes (Figures 6 and 7) were obtained, since the experimental points constraining the fit do not appear on the figure. Maybe the authors should represent the experimental points so that we can have a better idea of the match between the fit and the experiment.

We added a panel in Fig. 6B explaining the sigmoidal fits in detail.

4) Page 2, line 41: "... and significant variability results IN dysfunctional output".

Fixed.

5) Page 12, line 353: the terme slope factor is used in the text while the term scale factor is also to describe the same factor alpha. For clarity, the authors should chose one term and keep it throughout the manuscript.

Fixed.

## References

[B1] Akcay Z, Huang X, Nadim F, Bose A (2018) Phase-locking and bistability in neuronal networks with synaptic depression. Physica D 364:8–21. 10.1016/j.physd.2017.09.007 31462839 PMC6713463

[B2] Anwar H, Martinez D, Bucher D, Nadim F (2022) Inter-animal variability in activity phase is constrained by synaptic dynamics in an oscillatory network. eNeuro 9:1–19. 10.1523/ENEURO.0027-22.2022 35817566 PMC9319424

[B3] Asahina K, de Bivort BL, Grunwald Kadow IC, Yapici N (2022) Editorial: revisiting behavioral variability: what it reveals about neural circuit structure and function. Front Behav Neurosci 16:1–3. 10.3389/fnbeh.2022.956388 35783229 PMC9240743

[B4] Atamturktur S, Nadim F (2011) Effects of neuromodulator RPCH on short-term dynamics of a key synapse in an oscillatory network of crab *Cancer borealis*. J Appl Biol Sci 5:59–72.

[B5] Berens P (2009) Circstat: a MATLAB toolbox for circular statistics. J Stat Softw 31:1–21. 10.18637/jss.v031.i10

[B6] Bucher D, Prinz AA, Marder E (2005) Animal-to-animal variability in motor pattern production in adults and during growth. J Neurosci 25:1611–1619. 10.1523/JNEUROSCI.3679-04.2005 15716396 PMC6725924

[B7] Cerins A, Corp D, Opie G, Do M, Speranza B, He J, Barhoun P, Fuelscher I, Enticott P, Hyde C (2022) Assessment of cortical inhibition depends on inter individual differences in the excitatory neural populations activated by transcranial magnetic stimulation. Sci Rep 12:9923. 10.1038/s41598-022-14271-1 35705672 PMC9200840

[B8] Cronin EM, Schneider AC, Nadim F, Bucher D (2024) Modulation by neuropeptides with overlapping targets results in functional overlap in oscillatory circuit activation. J Neurosci 44:1–14. 10.1523/JNEUROSCI.1201-23.2023 37968117 PMC10851686

[B9] Daur N, Nadim F, Bucher D (2016) The complexity of small circuits: the stomatogastric nervous system. Curr Opin Neurobiol 41:1–7. 10.1016/j.conb.2016.07.005 27450880 PMC5270626

[B10] Dickinson PS, Powell DJ (2023) Diversity of neuropeptidergic modulation in decapod crustacean cardiac and feeding systems. Curr Opin Neurobiol 83:102802. 10.1016/j.conb.2023.102802 37922667

[B11] Doi A, Ramirez J-M (2008) Neuromodulation and the orchestration of the respiratory rhythm. Respir Physiol Neurobiol 164:96–104. 10.1016/j.resp.2008.06.007 18602029 PMC3606077

[B12] Feierstein CE, Portugues R, Orger MB (2015) Seeing the whole picture: a comprehensive imaging approach to functional mapping of circuits in behaving zebrafish. Neuroscience 296:26–38. 10.1016/j.neuroscience.2014.11.046 25433239

[B13] Garcia VJ, Daur N, Temporal S, Schulz DJ, Bucher D (2015) Neuropeptide receptor transcript expression levels and magnitude of ionic current responses show cell type-specific differences in a small motor circuit. J Neurosci 35:6786–6800. 10.1523/JNEUROSCI.0171-15.2015 25926455 PMC4412897

[B14] Goaillard J-M, Marder E (2021) Ion channel degeneracy, variability, and covariation in neuron and circuit resilience. Annu Rev Neurosci 44:335–357. 10.1146/annurev-neuro-092920-121538 33770451

[B15] Goaillard J-M, Taylor AL, Pulver SR, Marder E (2010) Slow and persistent postinhibitory rebound acts as an intrinsic short-term memory mechanism. J Neurosci 30:4687–4692. 10.1523/JNEUROSCI.2998-09.2010 20357119 PMC2885135

[B16] Goaillard J-M, Taylor AL, Schulz DJ, Marder E (2009) Functional consequences of animal-to-animal variation in circuit parameters. Nat Neurosci 12:1424–1430. 10.1038/nn.2404 19838180 PMC2826985

[B17] Golowasch J, Bose A, Guan Y, Salloum D, Roeser A, Nadim F (2017) A balance of outward and linear inward ionic currents is required for generation of slow-wave oscillations. J Neurophysiol 118:1092–1104. 10.1152/jn.00240.2017 28539398 PMC5547251

[B18] Golowasch J, Marder E (1992) Proctolin activates an inward current whose voltage dependence is modified by extracellular Ca^2+^. J Neurosci 12:810–817. 10.1523/JNEUROSCI.12-03-00810.1992 1347561 PMC6576042

[B19] Gray M, Daudelin DH, Golowasch J (2017) Activation mechanism of a neuromodulator-gated pacemaker ionic current. J Neurophysiol 118:595–609. 10.1152/jn.00743.2016 28446585 PMC5511868

[B20] Gray M, Golowasch J (2016) Voltage dependence of a neuromodulator-activated ionic current. eNeuro 3:1–19. 10.1523/ENEURO.0038-16.2016 27257619 PMC4874538

[B21] Gutierrez GJ, Marder E (2014) Modulation of a single neuron has state-dependent actions on circuit dynamics. eNeuro 1:1–12. 10.1523/ENEURO.0009-14.2014 26457324 PMC4596081

[B22] Hageter J, Waalkes M, Starkey J, Copeland H, Price H, Bays L, Showman C, Laverty S, Bergeron SA, Horstick EJ (2021) Environmental and molecular modulation of motor individuality in larval zebrafish. Front Behav Neurosci 15:777778. 10.3389/fnbeh.2021.777778 34938167 PMC8685292

[B23] Haldane JBS (1955) The measurement of variation. Evolution 9:484–484. 10.2307/2405484

[B24] Hamood AW, Haddad SA, Otopalik AG, Rosenbaum P, Marder E (2015) Quantitative reevaluation of the effects of short- and long-term removal of descending modulatory inputs on the pyloric rhythm of the crab, *Cancer borealis*. eNeuro 2:ENEURO.0058-14.2015. 10.1523/ENEURO.0058-14.2015 25914899 PMC4408878

[B25] Hamood AW, Marder E (2014) Animal-to-animal variability in neuromodulation and circuit function. Cold Spring Harb Symp Quant Biol 79:21–28. 10.1101/sqb.2014.79.024828 25876630 PMC4610821

[B26] Hamood AW, Marder E (2015) Consequences of acute and long-term removal of neuromodulatory input on the episodic gastric rhythm of the crab *Cancer borealis*. J Neurophysiol 114:1677–1692. 10.1152/jn.00536.2015 26156388 PMC4567610

[B27] Harris-Warrick RM (2011) Neuromodulation and flexibility in central pattern generator networks. Curr Opin Neurobiol 21:685–692. 10.1016/j.conb.2011.05.011 21646013 PMC3171584

[B28] Hentschke H (2011) abfload. *abfload*. https://www.mathworks.com/matlabcentral/fileexchange/6190-abfload. Accessed Nov. 30, 2020.

[B29] Khorkova O, Golowasch J (2007) Neuromodulators, not activity, control coordinated expression of ionic currents. J Neurosci 27:8709–8718. 10.1523/JNEUROSCI.1274-07.2007 17687048 PMC3558984

[B30] Li X, Bucher D, Nadim F (2018) Distinct co-modulation rules of synapses and voltage-gated currents coordinate interactions of multiple neuromodulators. J Neurosci 38:8549. 10.1523/JNEUROSCI.1117-18.2018 30126969 PMC6170985

[B31] Likhtik E, Johansen JP (2019) Neuromodulation in circuits of aversive emotional learning. Nat Neurosci 22:1586–1597. 10.1038/s41593-019-0503-3 31551602

[B32] Marder E (2012) Neuromodulation of neuronal circuits: back to the future. Neuron 76:1–11. 10.1016/j.neuron.2012.09.010 23040802 PMC3482119

[B33] Marder E, Bucher D (2007) Understanding circuit dynamics using the stomatogastric nervous system of lobsters and crabs. Annu Rev Physiol 69:291–316. 10.1146/annurev.physiol.69.031905.161516 17009928

[B34] Marder E, O’Leary T, Shruti S (2014) Neuromodulation of circuits with variable parameters: single neurons and small circuits reveal principles of state-dependent and robust neuromodulation. Annu Rev Neurosci 37:329–346. 10.1146/annurev-neuro-071013-013958 25032499

[B35] Marder E, Thirumalai V (2002) Cellular, synaptic and network effects of neuromodulation. Neural Netw 15:479–493. 10.1016/S0893-6080(02)00043-6 12371506

[B36] Marder E, Weimann JM (1992) Modulatory control of multiple task processing in the stomatogastric nervous system. In: *Neurobiology of motor programme selection: new approaches to mechanisms of behavioral choice* (Kien J, McCrohan C, Winlow W, eds), pp 3–19. Oxford: Pergamon.

[B37] McCormick DA, Nestvogel DB, He BJ (2020) Neuromodulation of brain state and behavior. Annu Rev Neurosci 43:391–415. 10.1146/annurev-neuro-100219-105424 32250724 PMC12237593

[B38] Moujaes F, Preller KH, Ji JL, Murray JD, Berkovitch L, Vollenweider FX, Anticevic A (2023) Toward mapping neurobehavioral heterogeneity of psychedelic neurobiology in humans. Biol Psychiatry 93:1061–1070. 10.1016/j.biopsych.2022.10.021 36715317

[B39] Nadim F, Bucher D (2014) Neuromodulation of neurons and synapses. Curr Opin Neurobiol 29:48–56. 10.1016/j.conb.2014.05.003 24907657 PMC4252488

[B40] Nassim C (2018) Good enough. In: *Lessons from the lobster: Eve Marder's work in neuroscience*. Cambridge, MA: The MIT Press.

[B41] Norris BJ, Wenning A, Wright TM, Calabrese RL (2011) Constancy and variability in the output of a central pattern generator. J Neurosci 31:4663–4674. 10.1523/JNEUROSCI.5072-10.2011 21430165 PMC3071692

[B42] Nusbaum MP, Blitz DM, Marder E (2017) Functional consequences of neuropeptide and small-molecule co-transmission. Nat Rev Neurosci 18:389–403. 10.1038/nrn.2017.56 28592905 PMC5547741

[B43] Nusbaum MP, Blitz DM, Swensen AM, Wood D, Marder E (2001) The roles of co-transmission in neural network modulation. Trends Neurosci 24:146–154. 10.1016/S0166-2236(00)01723-9 11182454

[B44] Palavicino-Maggio CB, Sengupta S (2022) The neuromodulatory basis of aggression: lessons from the humble fruit fly. Front Behav Neurosci 16:836666. 10.3389/fnbeh.2022.836666 35517573 PMC9062135

[B45] Ransdell JL, Nair SS, Schulz DJ (2013) Neurons within the same network independently achieve conserved output by differentially balancing variable conductance magnitudes. J Neurosci 33:9950–9956. 10.1523/JNEUROSCI.1095-13.2013 23761890 PMC6618398

[B46] Rihani K, Sachse S (2022) Shedding light on inter-individual variability of olfactory circuits in Drosophila. Front Behav Neurosci 16:835680. 10.3389/fnbeh.2022.835680 35548690 PMC9084309

[B47] Russo AF (2017) Overview of neuropeptides: awakening the senses? Headache 57:37–46. 10.1111/head.13084 28485842 PMC5424629

[B48] Schillaci MA, Schillaci ME (2022) Estimating the population variance, standard deviation, and coefficient of variation: sample size and accuracy. J Hum Evol 171:103230. 10.1016/j.jhevol.2022.103230 36115144

[B49] Schneider AC, Fox D, Itani O, Golowasch J, Bucher D, Nadim F (2021) Frequency-dependent action of neuromodulation. eNeuro 8:ENEURO.0338-21.2021. 10.1523/ENEURO.0338-21.2021 34593519 PMC8584230

[B50] Schneider AC, Itani O, Bucher D, Nadim F (2022) Neuromodulation reduces interindividual variability of neuronal output. eNeuro 9:ENEURO.0166-22.2022. 10.1523/ENEURO.0166-22.2022 35853725 PMC9361792

[B51] Stein W (2009) Modulation of stomatogastric rhythms. J Comp Physiol A 195:989–1009. 10.1007/s00359-009-0483-y 19823843

[B52] Swensen AM, Marder E (2000) Multiple peptides converge to activate the same voltage-dependent current in a central pattern-generating circuit. J Neurosci 20:6752–6759. 10.1523/JNEUROSCI.20-18-06752.2000 10995818 PMC6772805

[B53] Swensen AM, Marder E (2001) Modulators with convergent cellular actions elicit distinct circuit outputs. J Neurosci 21:4050–4058. 10.1523/JNEUROSCI.21-11-04050.2001 11356892 PMC6762692

[B54] Tamvacakis AN, Lillvis JL, Sakurai A, Katz PS (2022) The consistency of gastropod identified neurons distinguishes intra-individual plasticity from inter-individual variability in neural circuits. Front Behav Neurosci 16:855235. 10.3389/fnbeh.2022.855235 35309684 PMC8928192

[B55] Temporal S, Desai M, Khorkova O, Varghese G, Dai A, Schulz DJ, Golowasch J (2011) Neuromodulation independently determines correlated channel expression and conductance levels in motor neurons of the stomatogastric ganglion. J Neurophysiol 107:718–727. 10.1152/jn.00622.2011 21994267 PMC3349629

[B56] Thirumalai V, Prinz AA, Johnson CD, Marder E (2006) Red pigment concentrating hormone strongly enhances the strength of the feedback to the pyloric rhythm oscillator but has little effect on pyloric rhythm period. J Neurophysiol 95:1762–1770. 10.1152/jn.00764.2005 16319213

[B57] Tobin A-E, Cruz-Bermúdez ND, Marder E, Schulz DJ (2009) Correlations in ion channel mRNA in rhythmically active neurons. PLoS One 4:e6742. 10.1371/journal.pone.0006742 19707591 PMC2727049

[B58] Tran T, Unal CT, Severin D, Zaborszky L, Rotstein HG, Kirkwood A, Golowasch J (2019) Ionic current correlations are ubiquitous across phyla. Sci Rep 9:1687. 10.1038/s41598-018-38405-6 30737430 PMC6368568

[B59] Tsodyks M, Uziel A, Markram H (2000) Synchrony generation in recurrent networks with frequency-dependent synapses. J Neurosci 20:RC50. 10.1523/JNEUROSCI.20-01-j0003.2000 10627627 PMC6774142

[B60] Wenning A, Norris BJ, Doloc-Mihu A, Calabrese RL (2014) Variation in motor output and motor performance in a centrally generated motor pattern. J Neurophysiol 112:95–109. 10.1152/jn.00856.2013 24717348 PMC4064392

[B61] Wenning A, Norris BJ, Günay C, Kueh D, Calabrese RL (2018) Output variability across animals and levels in a motor system. eLife 7:e31123. 10.7554/eLife.31123 29345614 PMC5773184

[B62] Xu J, Lopez AJ, Hoque MM, Borich MR, Kesar TM (2022) Temporal profile of descending cortical modulation of spinal excitability: group and individual-specific effects. Front Integr Neurosci 15:777741. 10.3389/fnint.2021.777741 35197831 PMC8859157

[B63] Yuste R (2015) From the neuron doctrine to neural networks. Nat Rev Neurosci 16:487–497. 10.1038/nrn3962 26152865

[B64] Zhao S, Sheibanie AF, Oh M, Rabbah P, Nadim F (2011) Peptide neuromodulation of synaptic dynamics in an oscillatory network. J Neurosci 31:13991–14004. 10.1523/JNEUROSCI.3624-11.2011 21957260 PMC3407466

[B65] Zolin A, Cohn R, Pang R, Siliciano AF, Fairhall AL, Ruta V (2021) Context-dependent representations of movement in Drosophila dopaminergic reinforcement pathways. Nat Neurosci 24:1555–1566. 10.1038/s41593-021-00929-y 34697455 PMC8556349

