# Supplementary figures and images for: Convergent Comodulation Reduces Interindividual Variability of Circuit Output
Source: eNeuro. 2024 Sep 3;11(9):ENEURO.0167-24.2024. doi: 10.1523/ENEURO.0167-24.2024 (PMC11403100; doi:10.1523/ENEURO.0167-24.2024)

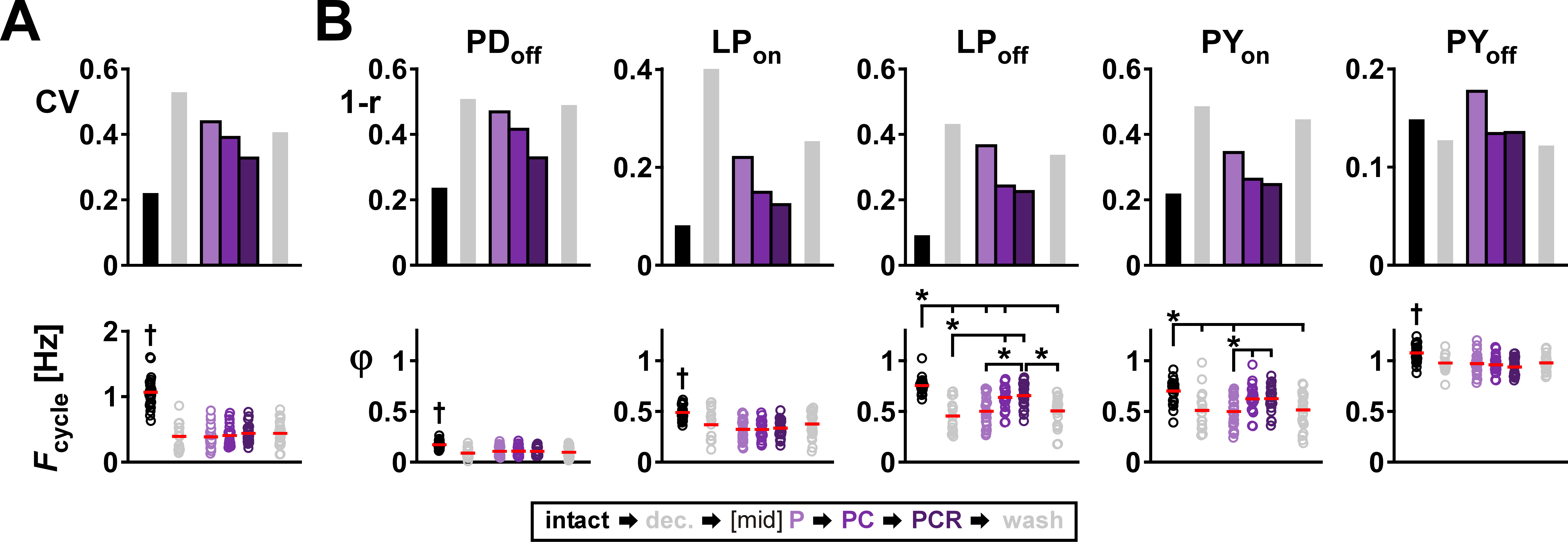

Supplement: Figure 3-2 — Comodulation at mid concentrations reduces the interindividual variability of rhythm parameters on the circuit output level in an independent dataset. (A) Fcycle and the corresponding CV under different modulatory conditions. (B) Burst start (on) and termination (off) and the corresponding circular variance, 1 - r, see Methods) under different modulatory conditions (color coded). N = 25 animals. Individual dots represent data from individual experiments, red bars indicate the (circular) mean. Asterisks indicate pairwise significant differences between two groups, or the group indicated with the longer line and those indicated with shorter lines (Dunn’s post-hoc test, p ≤ 0.05). The groups indicated with daggers are significantly different from all other groups but not from one another in that panel. All other pairwise comparisons were not statistically significant. Statistical results in Table 2. Download Figure 3-2, TIF file. [file eneuro-11-ENEURO.0167-24.2024-s010.tif]

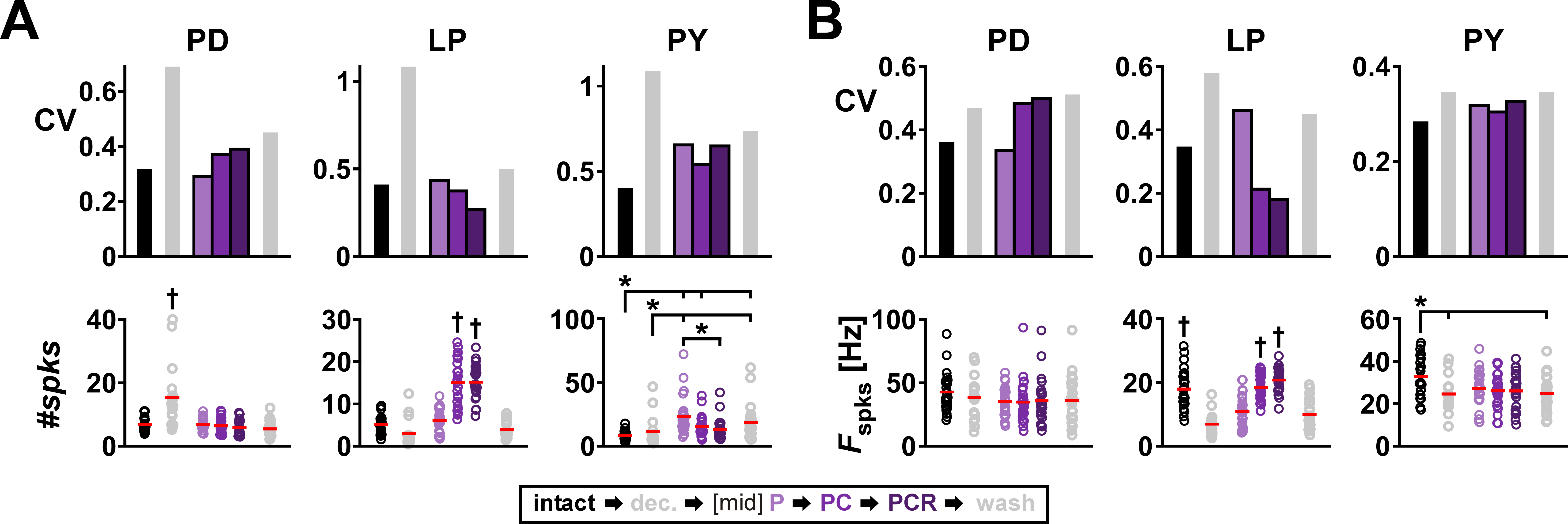

Supplement: Figure 4-2 — Comodulation at mid concentrations reduces the interindividual variability of rhythm parameters on the circuit output level in an independent dataset. (A) Average number of spikes (#spks) per burst and corresponding CV for each type of neuron at each modulatory condition (color coded). (B) Average spike frequency (Fspks) within a burst and corresponding CV for each type of neuron at each modulatory condition (color coded). N = 25 animals. Individual dots represent data from individual experiments, red bars indicate the (circular) mean. Asterisks indicate pairwise significant differences between two groups, or the group indicated with the longer line and those indicated with shorter lines (Dunn’s post-hoc test, p ≤ 0.05). The groups indicated with daggers are significantly different from all other groups in that panel. All other pairwise comparisons were not statistically significant. Statistical results in Table 2. Download Figure 4-2, TIF file. [file eneuro-11-ENEURO.0167-24.2024-s011.tif]
